# Hydrostaticity-Sensitive Structural Phase Transition and High-Pressure Phase Diagram in Fluorite: Evidence of Raman Spectroscopy and Electrical Conductivity

**DOI:** 10.3390/molecules31122078

**Published:** 2026-06-13

**Authors:** Mingyu Wu, Lidong Dai, Haiying Hu, Wenqing Sun, Meiling Hong, Chuang Li

**Affiliations:** 1 Key Laboratory of High-Temperature and High-Pressure Study of the Earth’s Interior, Institute of Geochemistry, Chinese Academy of Sciences, Guiyang 550081, China; wumingyu@mail.gyig.ac.cn (M.W.); hongmeilin@mail.gyig.ac.cn (M.H.); lichuang@mail.gyig.ac.cn (C.L.); 2 University of Chinese Academy of Sciences, Beijing 100049, China; 3 School of Physics and Electronic Science, Guizhou Normal University, Guiyang 550025, China; sunwenqing@gznu.edu.cn

**Keywords:** fluorite, Raman spectroscopy, electrical conductivity, phase transition, hydrostaticity, high temperature and high pressure

## Abstract

Raman spectroscopic analysis of fluorite was conducted in a diamond anvil cell (DAC) over a pressure range of 0.5–20.5 GPa under different hydrostatic environments, whereas the electrical conductivity was measured at 298–873 K and 1.2–19.6 GPa. High-resolution transmission electron microscopy (HRTEM) observations were performed on both the initial and recovered samples after recovery to ambient conditions. Three representative pressure-transmitting media (PTMs), including silicone oil, the mixture of methanol and ethanol (4:1 volume ratio, ME), and helium, were employed to control the degree of hydrostaticity within the DAC sample chamber. Experimental results indicate that the pressure-induced abrupt change in A_1g_, A_3g_, B_1g_ and B_2g_ Raman modes, together with the discontinuities in pressure-dependent Raman shifts, Grüneisen parameters, and electrical conductivity, can efficiently characterize the *α* (cubic structure, space group $Fm\bar{3}m$, No 225)-to-*γ* (cotunnite structure, PbCl_2_-type, space group *Pnma*, No 62) phase transition in fluorite. The transition pressures are determined to be 10.4, 9.6, 8.9 and 7.5 GPa under conditions of no PTM, silicone oil, ME and helium, respectively, demonstrating that the structural phase transition of fluorite is highly sensitive to hydrostaticity. Raman spectroscopy and electrical conductivity measurements upon decompression reveal that the phase transition is reversible, which is further confirmed by the HRTEM microstructural observation on both the initial and recovered samples. The linear relationships between electrical current and sinusoidal voltage, with the nonlinearity factors close to 1.00, manifest the Ohmic response of fluorite under high pressure. Finally, our high-temperature and high-pressure electrical conductivity results revealed the negative dependence of transition temperature on pressure, and the phase boundary between cubic and PbCl_2_-type fluorite was determined as: *P* (GPa) = 13.057 (±1.008) − 0.008 (±0.001) *T* (K). The obtained phase diagram of fluorite can be employed to deeply explore the high-pressure phase stability and structural transitions of other similar binary halide family minerals.

## 1. Introduction

As an important end-member of binary alkaline-earth metal fluorides, fluorite (CaF_2_) commonly occurs in low- to moderate-temperature hydrothermal deposits [1,2,3]. Particularly in deposits associated with the mineralization of metals (i.e., Cu, Pb, Zn, Au, etc.), it can be found in association with sulfide minerals such as pyrite (FeS_2_), galena (PbS), and chalcopyrite (CuFeS_2_), as well as carbonate minerals including calcite (CaCO_3_) and dolomite [CaMg(CO_3_)_2_] [4,5,6]. Under ambient conditions, fluorite crystallizes in the cubic structure (space group $Fm\bar{3}m$), where each calcium atom is coordinated by eight fluorine atoms, forming a highly symmetrical polyhedral framework. Owing to its distinctive physicochemical properties and widespread occurrence in ore-forming systems, fluorite has been extensively used as a geochemical indicator for tracing hydrothermal mineralization processes, particularly in rare earth element (REE)-enriched systems [7,8,9].

As a typical fluorine-bearing halide mineral with an AB_2_-type structure, fluorite has been extensively investigated under high-temperature and high-pressure conditions using Raman spectroscopy, whereas most previous studies were conducted using only a single pressure-transmitting medium [10,11,12]. For example, Kourouklis and Anastassakis investigated the phase transition of fluorite by Raman spectroscopy using ME as a pressure-transmitting medium over the pressure range of 0–12.0 GPa and room temperature in DAC [10]. They reported that the cubic (*α*)-to-orthorhombic (*γ*) phase transition in CaF_2_ occurs at 8.5 GPa. Subsequently, Speziale and Duffy carried out single-crystal Raman measurements on fluorite up to 50 GPa and identified seven Raman modes within the wavenumber range of 160–440 cm^−1^ [11]. New Raman features associated with the orthorhombic phase first emerged at 8.7 GPa, whereas the characteristic Raman mode of the cubic phase disappeared completely above 11 GPa, indicating completion of the pressure-induced structural transition. More recently, Zhang et al. investigated the phase transition of fluorite under high-temperature and high-pressure conditions using Raman spectroscopy up to 55 GPa at 300–700 K using an externally heated DAC with argon as the pressure-transmitting medium [12]. Their results showed that the *α*-to-*γ* phase transition of CaF_2_ occurs at 9.6 GPa and room temperature, which was confirmed by the disappearance of the T_2g_ Raman peak and appearance of 14 new Raman peaks. Moreover, their high-temperature and high-pressure experiments on CaF_2_ revealed that the pressure of the transition from cubic to cotunnite phase reduced with increasing temperature, indicating the negative correlation between transition pressure and temperature. Numerous high-pressure studies have demonstrated that hydrostaticity within the DAC sample chamber can exert a pronounced influence on the phase transition behavior of minerals during in situ Raman measurements, including kaolinite [13], barite [14], siderite [15], etc. In particular, Klotz et al. systematically evaluated pressure inhomogeneity in DAC experiments using eleven different pressure-transmitting media and revealed substantial differences in hydrostatic performance up to 50 GPa [16]. Despite these advances, a systematic investigation of the hydrostaticity-dependent *α*-to-*γ* phase transition in fluorite under high-temperature and high-pressure conditions has not yet been reported.

On the other hand, the pressure-induced discontinuity in electrical conductivity has been widely recognized as an effective indicator of structural phase transitions in many minerals under high-temperature and high-pressure conditions [14,17,18,19,20]. Previous electrical conductivity measurements on fluorite mainly focused on the electrical conduction of fluoride ion at the limited pressure of 1.0 GPa and 473–923 K using the piston-cylinder apparatus [21]. However, the electrical transport behavior and phase stability of fluorite under high-temperature and high-pressure conditions remain poorly constrained. Therefore, electrical conductivity measurements over a broad temperature–pressure range are important for understanding the phase stability and structural transition behavior of fluorite.

In the present study, we systematically investigated the hydrostaticity-dependent phase transition behavior of fluorite using high-pressure Raman spectroscopy, high-temperature and high-pressure *AC* impedance spectroscopy, and HRTEM observations of both the initial and recovered samples. Different hydrostatic environments within the DAC sample chamber were achieved using silicone oil, a 4:1 methanol–ethanol mixture (ME), and helium as pressure-transmitting media. At each designated pressure point, the phase transition temperature of fluorite was precisely determined. Accordingly, a phase diagram of fluorite was established over the wide temperature range of 298–873 K and pressures up to 20.5 GPa.

## 2. Results and Discussion

### 2.1. Raman Spectroscopy Results at Atmospheric Temperature and High Pressure

**Figure 1:**
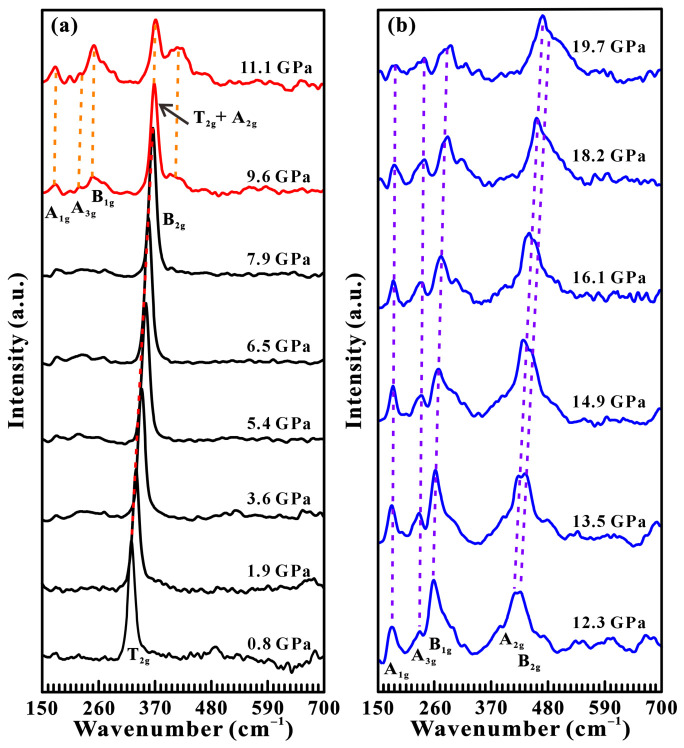
High-pressure Raman spectra of fluorite under non-hydrostatic conditions. (**a**) Spectra from 1.2 to 10.4 GPa and (**b**) from 11.9 to 20.5 GPa. Dashed lines indicate the pressure-induced shifts of the Raman peaks.

**Figure 2:**
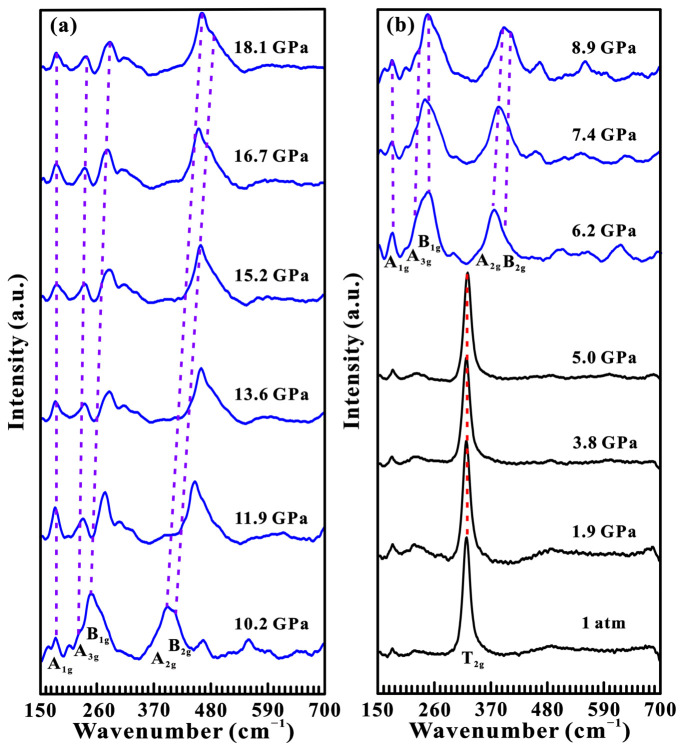
High-pressure Raman spectra of fluorite under non-hydrostatic conditions upon decompression. (**a**) Spectra from 18.1 to 10.2 GPa and (**b**) from 8.9 GPa to 1 atm. Dashed lines indicate the pressure-induced shifts of the Raman peaks.

Raman spectroscopy is a non-destructive and non-contact technique for detecting pressure-induced structural transitions in minerals under extreme conditions [22]. Figure 1 presents the Raman spectra of fluorite collected at 1.2–20.5 GPa and room temperature over the wavenumber range of 150–700 cm^−1^ under non-hydrostatic conditions. At 1.2 GPa, a single Raman characteristic peak is clearly identified at the position of 323.5 cm^−1^, which is assigned to the cubic T_2g_ mode of *α*-CaF_2_ [12,23,24]. This mode originates from the bending vibrations of F ions relative to Ca ions within the cubic fluorite framework [25]. With increasing pressure, the T_2g_ mode continuously shifts toward higher wavenumbers up to 10.4 GPa. At this pressure, five new Raman modes (*γ*-phase) emerge at the 173.6 (A_1g_), 224.5 (A_3g_), 261.9 (B_1g_), 426.7 (A_2g_), and 434.7 (B_2g_) cm^−1^, implying the onset of the *α*-to-*γ* phase transition in fluorite. Notably, the original cubic T_2g_ mode does not vanish immediately but overlaps with the A_2g_ mode. To further identify the overlapping vibrational modes during the phase-transition process, representative Raman spectra collected at key pressure points were deconvoluted using Gaussian functions, as shown in Figures S1 and S2. The Raman spectrum collected at the key pressure point of 10.4 GPa contains characteristic peaks of both phases (*α* and *γ*) (Figure S1a), which suggests transient two-phase coexistence in fluorite. As seen from Figure 1b, the phase transition is completed at 11.9 GPa, as evidenced by the complete disappearance of the cubic T_2g_ mode. Upon further compression to 20.5 GPa, all Raman peaks uniformly shift toward higher wavenumbers, accompanied by the progressive sharpening of the A_1g_, A_3g_, and B_1g_ modes, as well as the broadening of the A_2g_ mode, which can be attributed to the reduction in crystal symmetry under high pressure [26]. Upon decompression, all peaks shift back toward lower wavenumbers (Figure 2). When the pressure is released down to 5.0 GPa, the *γ*-phase Raman modes vanish, and the spectrum recovers to that of the initial cubic phase, demonstrating the reversibility of the pressure-induced transition. A pressure hysteresis of ~6.0 GPa is observed during decompression, which is likely related to the kinetic barriers to the structural transition upon decompression.

**Figure 3:**
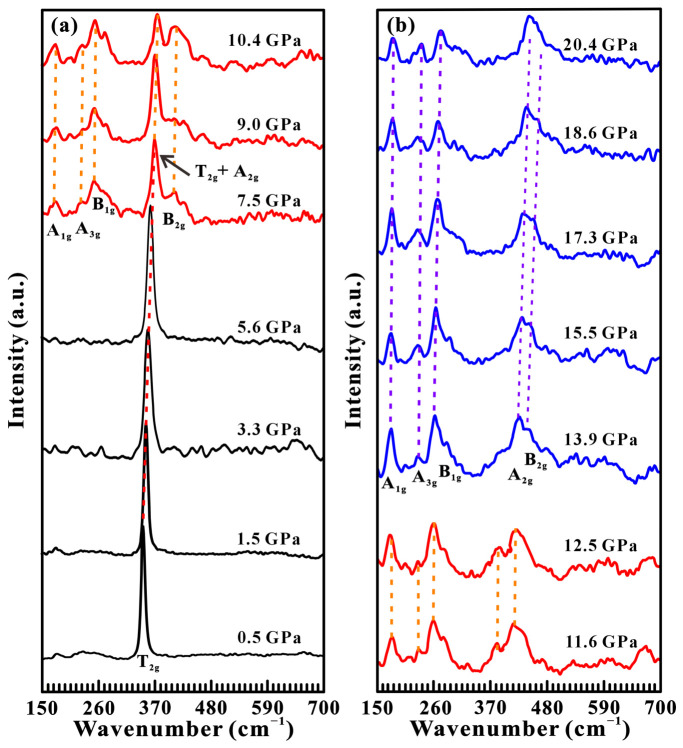
High-pressure Raman spectra of fluorite under hydrostatic conditions using helium as the pressure-transmitting medium. (**a**) Spectra from 0.5 to 10.4 GPa and (**b**) from 11.6 to 20.4 GPa. Dashed lines indicate the pressure-induced shifts of the Raman peaks.

**Figure 4:**
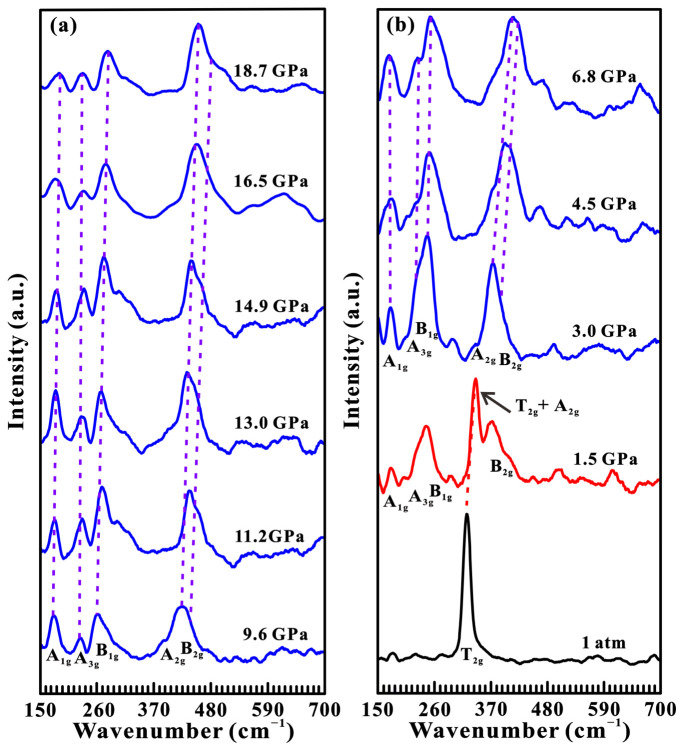
High-pressure Raman spectra of fluorite under hydrostatic conditions upon decompression using helium as the pressure-transmitting medium. (**a**) Spectra from 18.7 to 9.6 GPa and (**b**) from 6.8 GPa to 1 atm. Dashed lines indicate the pressure-induced shifts of the Raman peaks.

In order to evaluate the effect of hydrostaticity on the phase transition behavior of fluorite, three representative pressure-transmitting media (silicone oil, ME, and helium) were employed to generate various hydrostatic environments. The corresponding Raman spectra collected over 0.7–20.4 GPa are shown in Figure 3, Figures S3 and S4. When helium was employed as the pressure-transmitting medium, the *γ*-phase modes first appear at 7.5 GPa (Figure 3a), significantly lower than the transition onset observed under non-hydrostatic conditions (10.4 GPa). More importantly, the newly emerged *γ*-phase at 7.5 GPa modes exhibit substantially stronger intensities, indicating a stronger structural response under hydrostatic conditions. Notably, the cubic T_2g_ mode remains detectable up to 12.5 GPa (Figure 3b and Figure S1b), defining a broad coexistence interval between 7.5 and 12.5 GPa. Above 13.9 GPa, no residual Raman signal of the cubic T_2g_ mode is detected, indicating the completion of the phase transition. It is clear that the transition process under hydrostatic conditions is markedly more sluggish, as reflected by the broader two-phase coexistence pressure interval. Similar behaviors are observed for ME and silicone oil, where the transition initiates at 8.9 and 9.6 GPa, respectively, with coexistence intervals of 8.9–10.5 GPa and 9.6–11.1 GPa. These systematic variations demonstrate that the *α–γ* phase transition in fluorite is sensitive to the degree of hydrostaticity. The corresponding decompression process under hydrostatic conditions with different PTMs is presented in Figure 4, Figures S5 and S6 in detail. In helium, the *γ*-phase Raman modes persist down to ~1.6 GPa upon decompression (Figure 4). After released to ambient pressure, the Raman spectrum is reverted to its original state, further confirming the reversibility of the *α*-to-*γ* structural phase transition in fluorite under different hydrostatic environments. However, notable differences in the decompression process are observed under different hydrostatic environments. It makes clear that the orthorhombic *γ* phase persists until the pressure decreases to 3.0 GPa. Further decompressed to 1.5 GPa, Raman modes characteristic of both cubic (T_2g_) and orthorhombic (A_1g_, A_3g_, B_1g_, A_2g_ and B_2g_) structures coexist, with the corresponding Raman peaks located at 340.1, 173.8, 222.7, 245.8, 340.1, and 370.2 cm^−1^, respectively, indicating the coexistence of two phases upon decompression. The corresponding fitting results shown in Figure S2b further support the coexistence of the cubic and orthorhombic phases during decompression when using helium as the PTM. When ME and silicone oil are applied, the T_2g_ mode reappears at ~1.6–1.9 GPa, as displayed in Figures S5 and S6. Collectively, these results demonstrate a clear hysteresis effect in fluorite, which is significantly affected by the degree of hydrostaticity.

**Figure 5:**
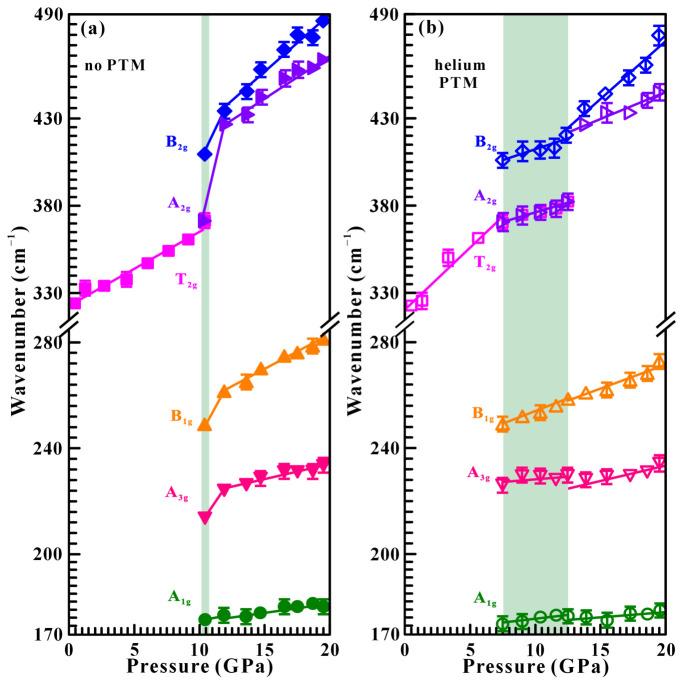
Pressure-dependent Raman shifts in fluorite under different hydrostatic environments: (**a**) non-hydrostatic condition and (**b**) hydrostatic condition using helium as the pressure-transmitting medium, respectively.

The pressure-dependent Raman shifts in fluorite, the corresponding linear fitting results (∂*ω*/∂*P*, cm^−1^ GPa^−1^) and Grüneisen parameter (*γ_i_*) values under different hydrostatic environments, are displayed and summarized in Figure 5 and Figure S7, as well as Table 1 and Table S1. Under non-hydrostatic conditions, the T_2g_ mode shifts toward higher wavenumbers with a steep and positive pressure coefficient of 4.23 cm^−1^ GPa^−1^ over 1.2–10.4 GPa. When pressure increases from 10.4 to 11.9 GPa, all Raman modes (A_1g_, A_3g_, B_1g_, A_2g_, T_2g_, and B_2g_) display systematic blueshift, with pressure coefficients of 0.70, 6.56, 8.90, 36.80, 36.80, and 15.86 cm^−1^ GPa^−1^, respectively. Notably, the slope of T_2g_ mode increases abruptly from 4.23 to 36.80 cm^−1^ GPa^−1^ and exhibits a clear inflection at 10.4 GPa, which indicates the onset of the *α*-to-*γ* phase transition in fluorite. Above 11.9 GPa, the T_2g_ mode disappears completely, and the remaining *γ*-phase modes continue to shift toward higher wavenumbers up to 20.5 GPa with relatively gentle pressure coefficients of 0.66, 1.15, 2.55, 6.62, and 5.02 cm^−1^ GPa^−1^, respectively. As shown in Figure 5a, the 2nd discontinuity at 11.9 GPa corresponds to the complete disappearance of the T_2g_ mode, proving the completion of the transition. Overall, these observable discontinuities in the pressure-dependent Raman shift within the critical interval of 10.4–11.9 GPa demonstrate that the *α*-to-*γ* structural phase transition in fluorite initiates at 10.4 GPa and completes at 11.9 GPa under non-hydrostatic conditions, which is in good agreement with the Raman spectroscopy results obtained above. In contrast, under hydrostatic conditions (helium, ME, and silicone oil), the evolution of Raman shifts is more sluggish, and the two-phase coexistence interval becomes significantly broader. Specifically, when helium is used as the PTM, the transition initiates at 7.5 GPa and completes at 12.5 GPa (Figure 5b), indicating a markedly more sluggish structural transformation compared to the non-hydrostatic case. Similar trends are observed for ME and silicone oil (Figure S7). These results demonstrate that the nucleation and growth of the high-pressure *γ* phase occur over a broader pressure range and are significantly affected by the degree of hydrostaticity.

In addition, the isothermal Grüneisen parameter (*γ_i_*) is a vital physical parameter in detecting structural phase transition in minerals, as it correlates the vibrational modes and elastic property at high pressure [27,28,29]. The isothermal *γ_i_* values of the corresponding Raman modes in fluorite were calculated,

(1) $$\gamma_{i}=-\;(\partial \;\ln\;\omega_{i})/(\partial \;\ln\;V)=-V/\omega_{i}\;(d\omega_{i}/dV)=K_{t}/\omega_{i}\;(d\omega_{i}/dP)$$

where *ω_i_* represents the Raman frequency of each mode, *V* stands for the unit-cell volume, d*ω*/d*P* is the pressure-dependent Raman shift, and the K_t_ is the isothermal bulk modulus of fluorite. As pointed out by Dorfman et al., the values of K_t0_ for the *α* and *γ* structural phases in fluorite were determined as 82 GPa and 74 GPa, respectively [30]. As listed in Table 1, under non-hydrostatic conditions, the characteristic T_2g_ mode exhibits a positive value (*γ_i_*) of 1.03 in the pressure range of 1.2–10.4 GPa, reflecting a typical hardening behavior of the T_2g_ mode under high pressure. At 10.4–11.9 GPa, the *γ_i_* value of the T_2g_ mode increases abruptly from 1.03 to 7.33. Meanwhile, the *γ_i_* value of five newly emerged Raman modes (A_1g_, A_3g_, B_1g_, A_2g_ and B_2g_) is calculated to be 0.29, 2.65, 7.33, 7.33, and 2.86, respectively. For the representative T_2g_ mode, this obvious jump in the *γ_i_* value at the critical pressure of 10.4 GPa clearly marks the onset of the *α*-to-*γ* structural phase transition of fluorite under non-hydrostatic conditions. Above 11.9 GPa, the higher-wavenumber modes (A_2g_ and B_2g_) have much larger *γ_i_* values compared to the lower-wavenumber modes (A_2g_, A_3g_ and B_1g_). This indicates that the A_2g_ and B_2g_ modes exhibit strong crystalline anharmonicity upon compression. Among five *γ*-phase Raman modes, the A_1g_ mode shows the smallest *γ_i_* value, suggesting relatively weak vibrational stability under high pressure. In contrast, when helium is used as the PTM, the *γ_i_* value of the T_2g_ mode shows an opposite trend, decreasing from 1.83 to 0.45 at 7.5 GPa, demonstrating the initiation of the phase transition under hydrostatic conditions. When ME and silicone oil were employed, similar phase transformations were observed at 8.9 and 9.6 GPa, respectively. In summary, although similar jumps in the *γ_i_* value of T_2g_ mode are observed under different hydrostatic environments, the magnitudes of *γ_i_* vary systematically, reflecting the significant influence of hydrostaticity on the elastic response and vibrational anharmonicity of fluorite during compression.

### 2.2. Electrical Conductivity Results at High Pressure

**Figure 6:**
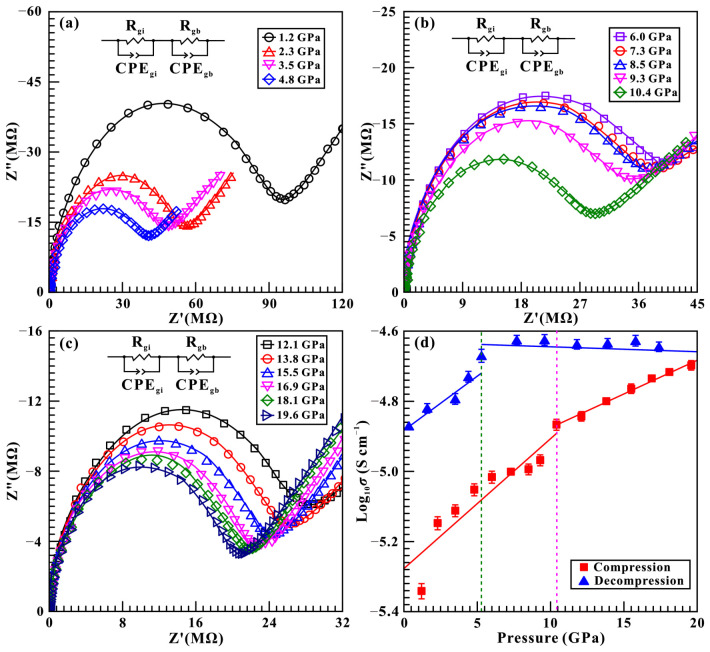
(**a**–**c**) Representative Nyquist diagram of impedance spectra of fluorite within the pressure range of 1.2–19.6 GPa. Z’ and Z″ represent the real and imaginary parts of the complex impedance, respectively. (**d**) Pressure-dependent electrical conductivity of fluorite upon compression and decompression. In this figure, solid and dashed lines serve as guides to the eye.

Representative Nyquist impedance spectra of fluorite were collected at a sinusoidal voltage of 1.0 V over the frequency range of 10^−1^–10^7^ Hz and are presented in Figure 6a–c. The acquired impedance spectra consist of a semicircular arc in the high-frequency region (10^3^–10^7^ Hz) and a tail in the low-frequency region (10^−1^–10^3^ Hz), which correspond to the grain interior resistance (*R*_gi_) and grain boundary resistance (*R*_gb_), respectively [31,32]. To accurately determine the resistance of fluorite, ZView software (V3.5) was utilized to process the acquired impedance spectra. The equivalent circuit model contains two series-connected elements, each comprising a resistor (R) connected in parallel with a constant phase element (CPE) [17,33]. The electrical conductivity (*σ*) of fluorite can be calculated using:

(2) σ = L/SR

where *L* (cm) and *S* (cm^2^) represent the thickness of the sample and cross-sectional area of the electrodes, respectively. Figure 6d shows the pressure dependence of logarithmic electrical conductivity of fluorite during compression and decompression. Below 10.5 GPa, the electrical conductivity of fluorite increases with a slope of 0.037 (±0.007) S cm^−1^ GPa^−1^. Above 10.5 GPa, the pressure dependence becomes less pronounced, with the slope decreasing to 0.019 (±0.001) S cm^−1^ GPa^−1^. Based on the change in the pressure dependence of electrical conductivity, the compression process can therefore be divided into two distinct pressure regions. This distinct change in slope at 10.5 GPa confirms the occurrence of the *α*-to-*γ* structural phase transition in fluorite, which is consistent with the Raman spectroscopic results (10.4 GPa) obtained above under non-hydrostatic conditions. In addition, minor conductivity anomalies are observed at approximately 2 and 5 GPa during compression. These low-pressure features are likely associated with the progressive closure of residual porosity within the pre-compressed powder sample, which may transiently influence electrical transport behavior. Importantly, these anomalies occur well below the structural transition pressure and therefore do not affect the identification of the *α*-to-*γ* phase transition. Upon decompression, the electrical conductivity remains nearly constant within the pressure range of 19.6–5.1 GPa. Upon further decompression to 0.5 GPa, the electrical conductivity decreases by approximately half an order of magnitude, which suggests that the pressure-induced structural transition in fluorite is reversible. A pronounced pressure hysteresis of ~ 6.0 GPa is observed, which is likely associated with the kinetic barriers to the structural transition during decompression.

**Figure 7:**
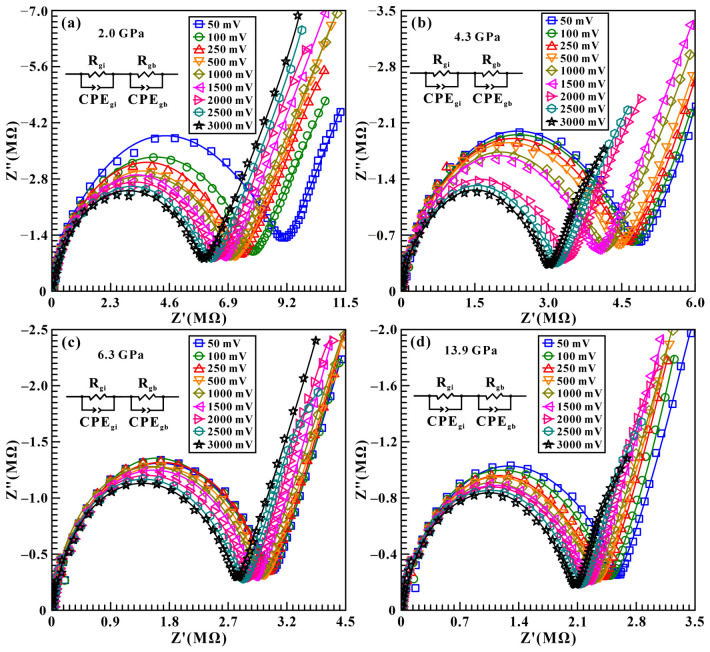
The Nyquist impedance spectra of fluorite over the sinusoidal voltage range of 50 to 3000 mV at four fixed pressures of (**a**) 2.0, (**b**) 4.3, (**c**) 6.3 and (**d**) 13.9 GPa. The colored curves represent the fitted impedance spectra.

**Figure 8:**
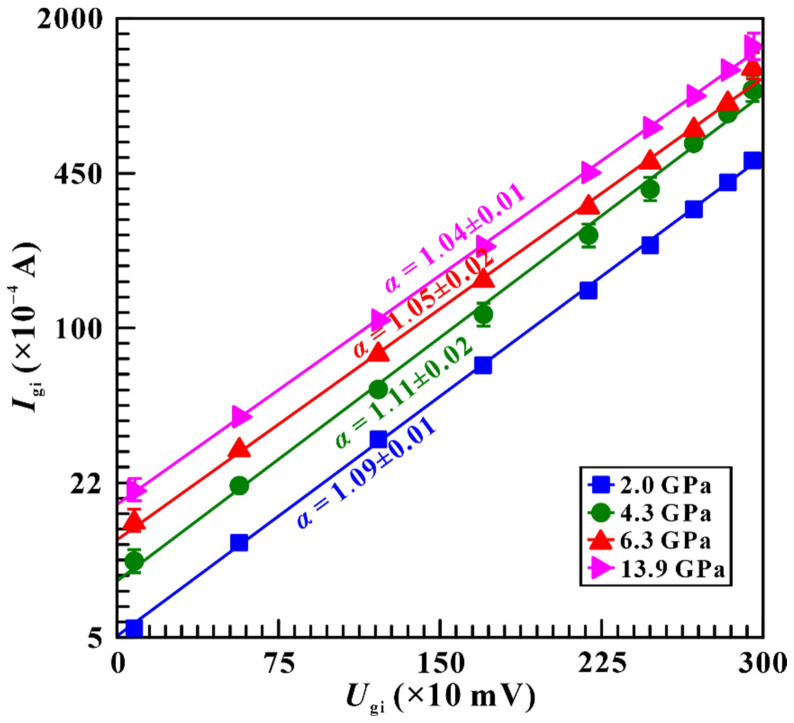
The dependence of electric current on the sinusoidal voltage in fluorite at four fixed pressures of 2.0, 4.3, 6.3 and 13.9 GPa.

In addition, impedance spectroscopy measurements performed under variable sinusoidal voltages can provide an effective means to characterize the pressure-induced Ohmic behavior of the sample at high pressure [34]. Nine sinusoidal voltages (50, 100, 250, 500, 1000, 1500, 2000, 2500, and 3000 mV) were applied at representative pressures of 2.0, 4.3, 6.3, and 13.9 GPa to systematically investigate the electrical transport behavior of fluorite across the structural phase transition. For instance, Nyquist impedance spectra collected at 2.0 GPa over a sinusoidal voltage range of 50–3000 mV are shown in Figure 7a. Using the equivalent circuit model described above, the electrical resistance of the sample was determined at each applied voltage, thereby establishing the relationship between electric current and sinusoidal voltage (Figure 8). Furthermore, the corresponding nonlinearity factor, defined as $\alpha \;=\;d\;(\mathrm{Log}\;I_{\mathrm{gi}})/d\;(\mathrm{Log}\;U_{\mathrm{gi}})$, was determined to be 1.09 ± 0.01 at 2.0 GPa, where *I*_gi_ and *U*_gi_ denote the grain-interior current and grain-interior bias voltage, respectively. Similarly, *α* values of 1.11 ± 0.02, 1.05 ± 0.02, and 1.04 ± 0.01 were obtained at 4.3, 6.3, and 13.9 GPa, respectively (Figure 7b–d). The near-unity values of α (≈1) across all measured pressures confirm the Ohmic behavior of fluorite under high pressure [34,35]. Similar Ohmic behavior has also been reported in UO_2_ single crystals, which crystallize in the same fluorite-type structure under ambient conditions [36].

### 2.3. The Microstructural Observation from the HRTEM Results

**Figure 9:**
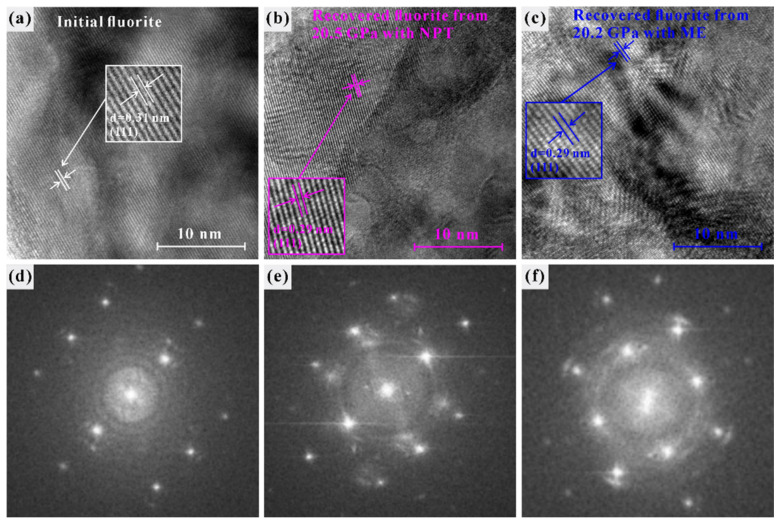
HRTEM and FFT micrographs of fluorite. Herein, (**a**,**d**) The initial sample; (**b**,**e**) The recovered sample decompressed from 20.5 GPa with NPT; (**c**,**f**) The recovered sample decompressed from 20.2 GPa using ME as the PTM. Here, the scale bar is 10 nm.

To further evaluate the reversibility of the pressure-induced structural transition under different hydrostatic environments, HRTEM observations were carried out on both the initial fluorite and the recovered samples obtained after decompression from 20.5 and 20.2 GPa under non-hydrostatic and hydrostatic conditions, respectively. Figure 9 presents representative HRTEM images together with the corresponding fast Fourier transform (FFT) patterns of the initial and recovered samples. As shown in Figure 9a, the initial fluorite exhibits clear and regularly arranged lattice fringes with an interplanar spacing of 0.31 nm, which can be indexed to the (111) crystallographic plane of cubic fluorite. The corresponding FFT pattern displays a series of well-defined diffraction spots (Figure 9d), indicating the high crystallinity of the initial sample. In contrast, the recovered samples still preserve well-resolved lattice fringes with an interplanar spacing of approximately 0.29 nm (Figure 9b–c), and the corresponding FFT patterns remain generally consistent with those of the pristine fluorite (Figure 9e–f). However, the recovered sample obtained under non-hydrostatic conditions exhibits a relatively higher degree of lattice distortion and diffraction spot broadening compared with that recovered under hydrostatic conditions, suggesting that the pressure-transmitting medium plays an important role in preserving crystalline integrity during compression and decompression processes. These HRTEM observations provide direct microstructural evidence for the reversibility of the *α*-to-*γ* structural transition in fluorite under different hydrostatic environments, in excellent agreement with the Raman spectroscopic and electrical conductivity results discussed above.

Three representative pressure media, including ME, silicone oil and helium together with NPT, were compared in detail, as summarized in Table 2. The *α*-to-*γ* structural transition pressure of fluorite increases progressively from 7.5 to 10.4 GPa in the sequence of helium, ME, silicone oil, and NPT conditions, clearly demonstrating the strong dependence of the phase transition behavior on hydrostaticity. Such variations are likely associated with differences in deviatoric stress within the DAC sample chamber under different hydrostatic environments. As pointed out by Klotz et al., various pressure-transmitting media exhibit markedly different solidification pressures, which directly influence the distribution of deviatoric stress during compression [16]. Similar hydrostaticity-sensitive phase transition behavior has also been reported in other binary fluorine-bearing alkaline-earth halides, such as barium fluoride (BaF_2_), which crystallizes in the same fluorite-type cubic structure (space group $Fm\bar{3}m$) as CaF_2_ [37]. Furthermore, combined evidence from Raman spectroscopy, electrical conductivity measurements, and HRTEM observations consistently demonstrates that the hydrostaticity-dependent *α*-to-*γ* structural transition in fluorite is reversible.

### 2.4. Phase Diagram of Fluorite at High Temperature and High Pressure

**Figure 10:**
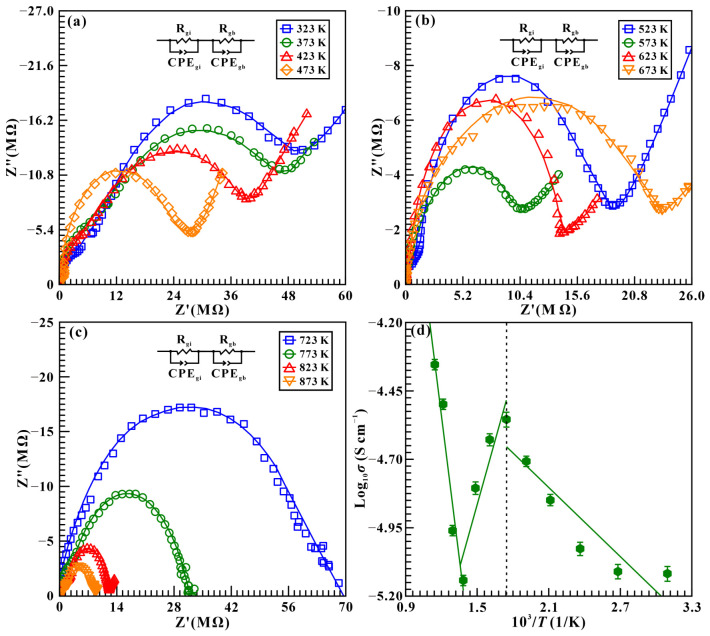
Representative Nyquist diagram of impedance spectra of fluorite measured at a given pressure of 7.0 GPa over the temperature range of 323–873 K. (**a**) 323–473 K; (**b**) 523–673 K; (**c**) 723–873 K; (**d**) the logarithmic electrical conductivity of sample against reciprocal temperature. Solid and dashed lines are provided as visual guides.

To construct the high-*T* and high-*P* phase diagram of fluorite, a series of electrical conductivity measurements were conducted at fixed pressures of 7.0, 8.1, and 9.3 GPa over the temperature range of 323–873 K, as depicted in Figure 10, Figure S8 and S9 in detail. Representative Nyquist impedance spectra collected at 7.0 GPa are presented in Figure 10a–c, and the corresponding logarithmic electrical conductivity as a function of reciprocal temperature is illustrated in Figure 10d. At 7.0 GPa, the electrical conductivity of fluorite exhibits a pronounced positive temperature dependence over 323–573 K and 723–873 K. By contrast, conductivity declines with rising temperature across the 573–723 K interval, showing an anomalous negative temperature dependence. Such anomalous electrical conductivity behavior is closely associated with the pressure-driven *α*-to-*γ* structural transition of fluorite. Specifically, the abrupt decrease in electrical conductivity at 573 K is interpreted as the onset of the cubic-to-PbCl_2_-type structural transition, whereas the transition is considered complete above 723 K. Accordingly, the onset temperature of the *α*-to-*γ* transition at 7.0 GPa is determined to be approximately 573 K. Similar conductivity anomalies are also observed at higher pressures (Figures S8 and S9). Based on these conductivity discontinuities, the *α*-to-*γ* structural transition temperatures of fluorite are determined to be 523 K and 473 K at 8.1 and 9.3 GPa, respectively.

**Figure 11:**
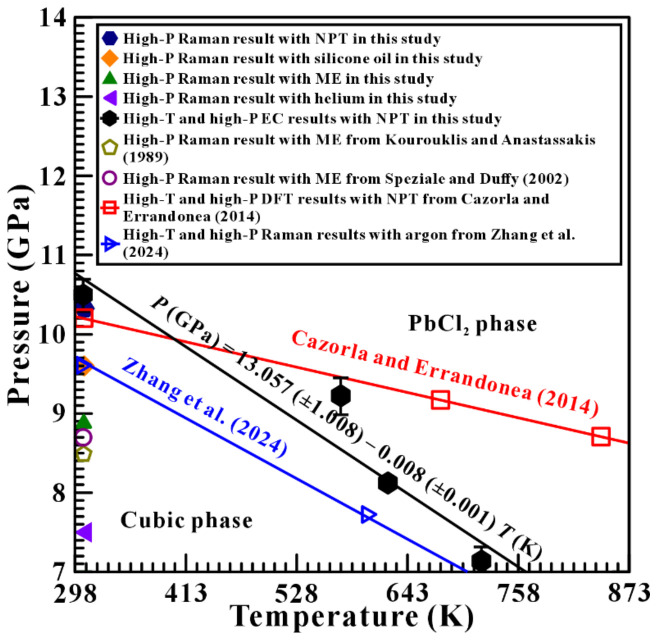
The proposed *P*–*T* phase diagram of fluorite investigated at temperatures of 298–873 K and pressures of 7.0–14.0 GPa. The data points correspond to the *α–γ* structural phase transition from refs. [10,11,12,38]. Black solid line represents the structural transition boundary of cubic fluorite to PbCl_2_ structure. The red and blue solid lines stand for the phase boundary between *α* (cubic fluorite structure) to *γ* (cotunnite structure) phase from refs. [12,38], respectively.

Based on the presented data of Raman spectroscopy and *AC* impedance measurements, the phase boundary between the cubic and PbCl_2_-type structures of fluorite can be expressed as,

(3) P (GPa)=13.057 (±1.008)−0.008 (±0.001) T (K)

where the signals of *P* and *T* stand for the experimental pressure (GPa) and temperature (K), respectively. Figure 11 shows the *P*–*T* phase diagram of fluorite over the temperature range of 298–873 K and pressure of 7.0–14.0 GPa, revealing a negative slope between *α* and *γ* phase transition (–0.008 GPaK^−1^). At room temperature, the hydrostaticity-dependent *α*-to-*γ* structural transition in fluorite was constrained by Raman spectroscopy and electrical conductivity measurements up to 20.5 GPa. Meantime, the electrical conductivity of fluorite at high-*T* and high-*P* conditions further reveals the occurrence of structural phase transformation at 298–873 K and 7.0–14.0 GPa. For comparison, the transition pressures reported in previous studies using single-crystal fluorite and different pressure-transmitting media are also plotted in Figure 11. The transition pressures obtained in the present study using powder samples are generally consistent with those reported for single-crystal samples, suggesting that the initial sample form exerts only a limited influence on the structural transition behavior of fluorite. As is known, the Clausius–Clapeyron relation (CCR, d*T*/d*P* = ∆V/∆S) is widely applied to describe the relationship among entropy change (∆S), molar volume change (∆V), and the slope of phase transition (d*T*/d*P*), which can well interpret the structural phase transition slope of fluorite at high temperature and high pressure [39]. Previous synchrotron X-ray diffraction studies demonstrated that the *α*-to-*γ* structural transition in fluorite is accompanied by an apparent volume contraction of approximately 11% [40], indicating a negative ΔV value. As pointed out by Boulfelfel et al., the *α*-to-*γ* structural phase transition of fluorite was identified as a gradually symmetry-lowering process due to the reorganization of one half of the octahedral voids [41], and thus, the ΔS is a positive value. Therefore, the Clapeyron slope (d*T*/d*P*) for the structural phase transition boundary of fluorite from cubic to PbCl_2_-type structure is a negative value, which agrees well with our electrical conductivity results under high-temperature and high-pressure conditions. Moreover, the slope obtained in our study (–0.008 GPaK^−1^) is steeper than those reported from first-principles calculations (–0.003 GPaK^−1^) [38] and previous high-*T* and high-*P* Raman scattering results (–0.006 GPaK^−1^) [12]. Such discrepancies are likely associated with the different approaches employed in previous studies.

### 2.5. Conduction Mechanism

It is well known that the relationship between electrical conductivity and reciprocal temperatures follows the Arrhenius equation,

(4) $$\sigma \;=\sigma_{0}\;\exp\;(-∆H/kT)$$

where *σ* is the electrical conductivity of sample (S/cm), *σ_0_* is the pre-exponential factor (S/cm), ∆*H* is the activation enthalpy (eV), *k* is the Boltzmann constant (eV/K), and *T* is the absolute temperature (K). It is general that the activation enthalpy derived from the Arrhenius relationship within a specific temperature–pressure range can provide critical constraints on the dominant conduction mechanism in minerals and rocks [42,43,44,45]. The corresponding fitting parameters for fluorite are summarized in Table 3. Based on the present results, the activation enthalpies range from 0.11 to 0.87 eV over 373–873 K at representative pressures of 7.0, 8.1, and 9.3 GPa. In fluorite-structure crystals, ionic conductivity is predominantly governed by the thermally activated migration of point defects via a hopping mechanism [46,47]. According to previous studies on the electrical conductivity of fluorite [21,46,48], ionic conduction is the dominant mechanism under high-temperature and high-pressure conditions, with fluorine ions serving as the primary charge carriers. At the microscopic scale, ionic conduction in solids is governed by the diffusion of charge carriers, and the relationship between diffusion and electrical conductivity can be described by the Nernst–Einstein equation [49],

(5) $$\sigma \;=Dcq^{2}\;/\;Hr\;k\;T$$

where *H_r_* is the Haven ratio, *D* is the diffusion coefficient, *c* is the charge carrier concentration, *q* is the electrical charge of the charged species, *k* is the Boltzmann constant, and *T* is the absolute temperature. Previous self-diffusion experiments on CaF_2_ single crystals have demonstrated that both Ca^2+^ and F^−^ migrate via a vacancy-mediated mechanism [50]. However, anion defects exhibit significantly higher mobility than cation defects, indicating that the contribution of Ca^2+^ to the overall ionic conductivity is negligible. Matzke also reported an activation enthalpy of ~0.90 eV for fluorine self-diffusion below 973 K, which is characteristic of extrinsic diffusion behavior [50]. On this basis, Oberschmidt and Lazarus proposed that ionic conduction can be classified as extrinsic vacancy conduction when the activation enthalpy is lower than ~0.90 eV [46]. The activation enthalpies obtained in the present study (0.11–0.87 eV) fall within this range, which demonstrates that fluorite is dominated by extrinsic vacancy-mediated ionic conduction over the investigated pressure range of 0.5–20.5 GPa and temperature range of 323–873 K. Importantly, the identical low activation enthalpies (<0.9 eV) observed before and after the *α–γ* structural phase transition suggest that the dominant conduction mechanism remains unchanged across the phase boundary. A transformation in conduction mechanism may occur with increasing temperature, changing from extrinsic vacancy conduction at relatively low temperatures to intrinsic conduction at higher temperatures [21]. However, the relevant transition temperature among these two mechanisms is higher than the temperature range investigated in this study, further supporting the dominance of extrinsic conduction under our experimental conditions. According to Boulfelfel et al. and Cazorla et al., the point defect in fluorite crystal is the anti-Frenkel defect [38,41]. Under high-temperature and high-pressure conditions, fluorine ions (F^−^) gain sufficient thermal energy to overcome the migration barrier and hop into adjacent interstitial sites, leaving behind vacancies in the anion sublattice and forming anti-Frenkel defect pairs. The defect reaction of fluorite can be described as,

(6) $$F_{F}^{x}\;⇌\;F_{i}^{\prime }\;+\;V_{F}^{\prime }$$

where $F_{F}^{x}$ denotes a fluorine ion at a regular lattice site, $F_{i}^{\prime }$ represents a negatively charged interstitial fluorine ion, and $V_{F}^{\prime }$ corresponds to a positively charged fluorine vacancy. These mobile fluorine vacancies are therefore responsible for the observed ionic conductivity in fluorite.

### 2.6. First-Principles Theoretical Calculations at High Pressure

**Figure 12:**
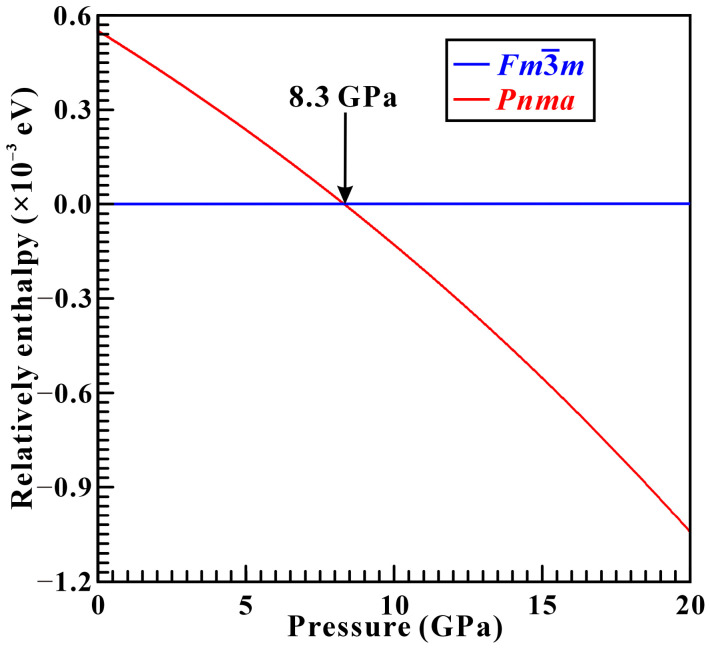
The enthalpy difference between $Fm\bar{3}m$ and *Pnma* phases in fluorite as a function of pressure within the pressure range of 0–20.0 GPa.

To further substantiate the pressure-induced structural transition identified by Raman spectroscopy and electrical conductivity measurements, first-principles calculations were performed for CaF_2_ under high pressure. The transition pressure was determined from calculated enthalpy differences between the cubic fluorite ($Fm\bar{3}m$) and orthorhombic (*Pnma*) phases over the pressure range of 0–20 GPa. As shown in Figure 12, the $Fm\bar{3}m$ phase possesses lower enthalpy than the *Pnma* phase below 8.3 GPa, indicating that the cubic structure is thermodynamically stable in this pressure interval. Above 8.3 GPa, the *Pnma* phase becomes energetically favorable, marking the *α–γ* transition at ~8.3 GPa. This theoretical transition pressure is in reasonable agreement with the experimentally determined transition pressure (~10.5 GPa under non-hydrostatic conditions). The modest discrepancy is expected because first-principles calculations assume ideal, stress-free crystalline structures at 0 K, whereas the experiments were conducted on natural samples under non-hydrostatic conditions and room temperature.

**Figure 13:**
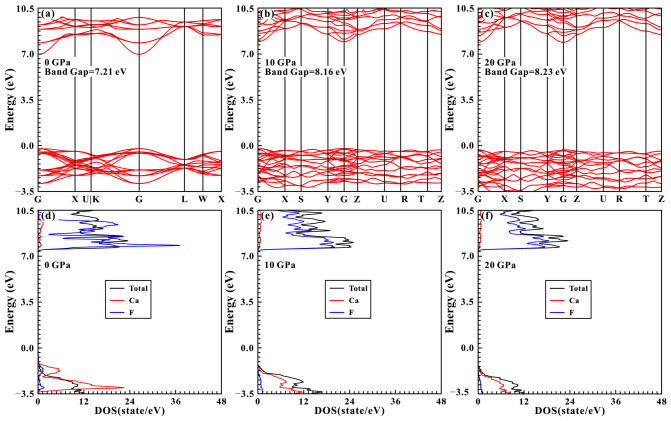
Calculated electronic band structures (**a**–**c**) and their corresponding density of states (**d**–**f**) for CaF_2_ at three representative pressures of (**a**,**d**) 0 GPa, (**b**,**e**) 10.0 GPa, and (**c**,**f**) 20.0 GPa, respectively.

Based on the optimized geometries, the theoretical structural parameters of CaF_2_ at 0, 10, and 20 GPa are summarized in Table S2, while the corresponding electronic band structures and density of states are displayed in Figure 13a–f. At ambient pressure, the optimized lattice constant of the $Fm\bar{3}m$ phase is a = 5.521 Å, which is approximately 1.1% larger than the experimental value of 5.463 Å [51]. This slight overestimation is consistent with the well-known tendency of GGA functionals to slightly overestimate lattice constants. At 10 and 20 GPa, the optimized structural parameters correspond to the *Pnma* phase, which is consistent with the calculated phase-transition pressure of ~8.3 GPa. With increasing pressure, all lattice constants of the *Pnma* phase decrease monotonically, reflecting the continuous compression of the unit cell. The calculated electronic band structures (Figure 13a–c) further show that both the valence band maximum and conduction band minimum are located at the Γ point under ambient conditions, indicating that CaF_2_ is a direct band-gap insulator. The calculated band gap of 7.21 eV agrees well with previous theoretical results reported by Cui et al. [52]. With increasing pressure, the overall band dispersion slightly increases and the band gap widens to 8.16 eV at 10 GPa and 8.23 eV at 20 GPa. The valence band maximum remains located at Γ, preserving the direct-gap character throughout the investigated pressure range. The total and partial density of states (Figure 13d–f) reveal that the valence band is dominated by F 2p states, whereas the conduction band minimum primarily originates from Ca 3d states. The relatively weak hybridization between these states confirms the strongly ionic nature of CaF_2_. Under compression, both the valence and conduction bands exhibit moderate broadening, consistent with the enhanced band dispersion observed in the electronic band structures. Importantly, CaF_2_ remains a wide-band-gap insulator throughout the investigated pressure range. The pressure-induced widening of the band gap further suppresses electronic excitation, indicating that electronic carriers contribute negligibly to electrical transport. Instead, electrical conductivity at high temperature and pressure is mainly governed by defect-mediated ionic migration, in good agreement with the conduction mechanism inferred from our experimental results.

## 3. Experimental Procedures

### 3.1. Sample Preparation

**Figure 14:**
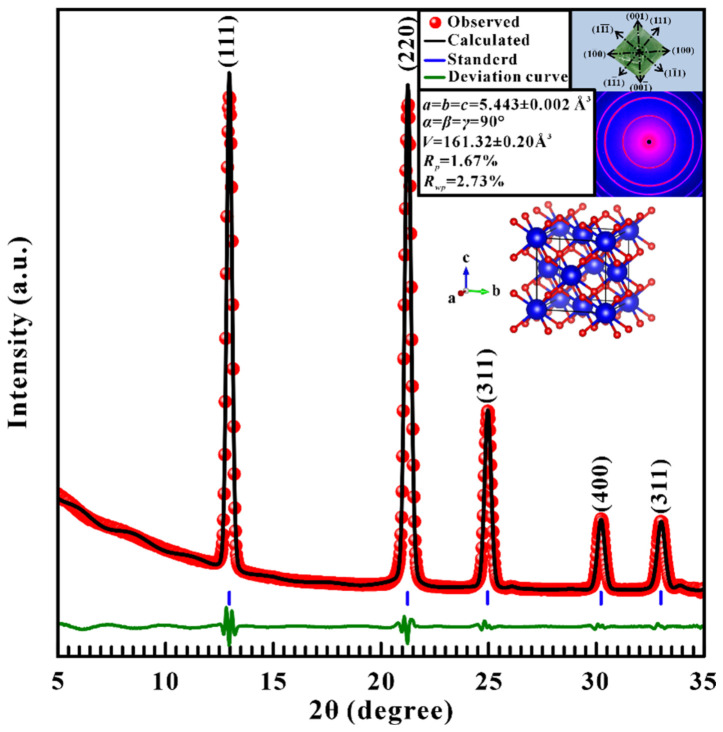
The powder XRD pattern and structural refinement of fluorite under ambient conditions. Inset: The optical microscope image and the calculated lattice parameters for the initial sample. The red solid circles and its correspondent black lines stand for the Rietveld fittings for the observed and calculated results, respectively. Blue vertical bars represent the standardized positions of Bragg peaks. The green solid line represents the observed and calculated deviation curve. Each diffraction peak is labeled with its corresponding Miller indices.

In this study, the fluorite single crystal was gathered from Hengnan County in Hunan province, China. The single crystal sample was polished and crushed into a fine powder using an agate mortar. The sample was characterized by a Rigaku SmartLab-type X-ray diffractometer (Rigaku Corporation, Tokyo, Japan) with Mo K*α* radiation (*λ* =  0.7107 Å) operating at 20 mA and 40 kV, scanning a 2*θ* range of 3–40° in the Key Laboratory of High-Temperature and High-Pressure Study of the Earth’s Interior, Institute of Geochemistry, Chinese Academy of Sciences. Subsequently, the obtained XRD data were analyzed using the Rietveld refinement program using the General Structure Analysis System (GSAS-II) software (V5831) package. As shown in Figure 14, all of the acquired diffraction peaks can be indexed to the cubic fluorite with the space group of $Fm\bar{3}m$, and the correspondent lattice parameters were refined as *a* = *b* = *c* = 5.443 ± 0.002 Å, *α* = *β* = *γ* = 90° and *V* = 161.32 ± 0.20 Å^3^, which are in good agreement with previous data [53,54].

### 3.2. High-Pressure Raman Spectroscopy Measurements

A symmetric DAC with a 200 μm anvil culet and 8.5° bevel angle was adopted for high-pressure Raman scattering experiments of fluorite up to 20.5 GPa. The T-301 stainless-steel gasket was pre-indented to a thickness of 50 µm at around 10.0 GPa, after which a 100 µm-diameter hole was laser-drilled to serve as the sample chamber. Powder samples were utilized to explore the effect of hydrostaticity on the structural phase transition behavior of fluorite. Prior to the high-pressure Raman spectroscopy measurements, the experimental samples were dried in a vacuum oven at 373 K for 24 h to remove adsorbed water. Pre-compressed fluorite powder pellets together with a small ruby ball (grain size of ~5 μm) were loaded into the sample chamber. Pressure was calibrated using the wavenumber shift in the Cr^3+^ fluorescence band from ruby [55]. Three pressure-transmitting media, including silicone oil, ME and helium, were used to realize different hydrostatic environments in the DAC sample chamber, whereas no pressure-transmitting medium was applied to obtain the non-hydrostatic condition. High-pressure Raman spectra of fluorite were collected using the Renishaw inVia Raman microscope in the backscattering configuration (Renishaw plc, Gloucestershire, UK) equipped with the 1800 lines/mm diffraction grating and an Ar^+^ laser (514.5 nm excitation mode). Raman signals were collected through a 20× objective lens (numerical aperture NA = 0.40), corresponding to an approximate laser spot size of ~2–3 μm on the sample surface. The laser power at the sample surface was maintained at 50 mW with an acquisition time of 240 s for each spectrum. The 514.5 nm excitation wavelength was selected because it provides a strong Raman response for CaF_2_ while minimizing fluorescence background interference. In addition, the selected laser power was adopted to minimize possible local heating effects within the DAC sample chamber during high-pressure measurements. Each acquired Raman spectrum was baseline-corrected, and the Raman peaks were fitted using a Gaussian peak profile in Peakfit software (V4.12) to extract the peak positions.

### 3.3. High-Temperature and High-Pressure Electrical Conductivity Measurements

High-temperature and high-pressure electrical conductivity measurements were conducted in a four-column-type DAC over the wide temperature range of 298–873 K and 0.5–20.5 GPa. The T-301 stainless-steel gasket was pre-indented to a final thickness of ~50 μm at ~10.0 GPa. A hole of 180 μm in diameter was subsequently drilled at the center of the indentation using a laser drilling system. The mixture of boron nitride and epoxy powder was then tightly packed into the hole and compacted up to ~15.0 GPa to electrically insulate the electrodes from the metallic gasket. A central hole of 100 μm in diameter was then fabricated as the insulating sample chamber. To ensure adequate electrical insulation between the two symmetric electrode leads and the metallic gasket, the remaining gasket surface was coated with the insulating cement. Two platinum electrodes were symmetrically placed on the upper and lower sides of the sample chamber. High temperature was generated by double external electrical resistance furnaces tightly wrapped around the tungsten carbide base. At each target temperature, a sufficient equilibration time of ~30 min was maintained to ensure thermal equilibrium between the anvils and the sample chamber, during which both the temperature readout and impedance spectra remained stable without appreciable fluctuation. The temperature was precisely calibrated using a *k*-type thermocouple adhered to the side face of the diamond anvil, with a temperature uncertainty of ±5 K. No pressure-transmitting medium was applied to avoid the introduction of additional impurities and to ensure good contact between electrodes and sample. The experimental assemblage for the high-temperature and high-pressure electrical conductivity measurements is schematically illustrated in Figure S10. The *AC* impedance spectroscopy of fluorite was carried out at 298–873 K and 0.5–19.6 GPa using the Solartron-1260 impedance spectroscopy analyzer (Solartron Analytical, Farnborough, UK) over the wide frequency range of 10^−1^–10^7^ Hz. The detailed measurement principles and experimental procedures are described in our previous works [56].

### 3.4. First-Principles Theoretical Calculations

First-principles theoretical calculations on CaF_2_ were performed using the Cambridge Sequential Total Energy Package (CASTEP) code within the Material Studio package based on density functional theory (DFT). The electronic exchange–correlation interactions were treated within the generalized gradient approximation (GGA) using the Perdew–Burke–Ernzerhof (PBE) functional [57,58]. The kinetic energy cutoff was set to 650 eV. The Brillouin zone was sampled using Monkhorst–Pack k-point meshes of 8 × 8 × 8 and 4 × 7 × 4 for the $Fm\bar{3}m$ and *Pnma* structures, respectively [59]. Structural optimizations were carried out prior to the electronic structure calculations. The relative stability of the two structural phases was evaluated by comparing their enthalpies calculated from H = E + PV over the pressure range of 0–20 GPa. In addition, the electronic band structures and density of states of CaF_2_ were calculated at 0, 10, and 20 GPa to investigate the pressure-dependent electronic properties of fluorite.

## 4. Conclusions

In this study, the hydrostaticity-dependent structural phase transition behavior of fluorite was systematically investigated using Raman spectroscopy under different pressure-transmitting environments, combined with high-temperature and high-pressure electrical conductivity measurements and HRTEM observations. The Raman results demonstrate that the *α*-to-*γ* structural phase transition in fluorite is highly sensitive to hydrostaticity. The transition pressure decreases systematically from 10.4 GPa under non-hydrostatic conditions to 7.5 GPa under helium medium, accompanied by pronounced differences in the pressure interval of two-phase coexistence. These observations indicate that deviatoric stress plays an important role in regulating the phase-transition behavior of fluorite under compression. Combined Raman spectroscopy, electrical conductivity measurements, and HRTEM observations consistently confirm that the *α*-to-*γ* structural phase transition in fluorite is reversible during decompression under different hydrostatic environments. In addition, electrical conductivity measurements further constrain the phase boundary of fluorite between cubic and PbCl_2_-type structures over 298–873 K and 7.0–14.0 GPa, yielding a negative Clapeyron slope described by: P (GPa) = 13.057 (±1.008)−0.008 (±0.001) T (K). The activation energies obtained under high-temperature and high-pressure conditions indicate that fluorite is dominated by extrinsic vacancy-mediated fluorine-ion conduction throughout the investigated temperature–pressure range. Overall, the present results provide new insights into the hydrostaticity-sensitive phase transition behavior and transport properties of fluorite, with broader implications for understanding phase stability and structural transitions in fluorite-type halide materials under extreme conditions.

## Figures and Tables

**Table 1:** Pressure-dependent Raman shift (d*ω*/d*P*, cm^−1^ GPa^−1^) for fluorite under different hydrostatic environments. Here, *ω* (cm^−1^) and *P* (GPa) represent the Raman wavenumber and pressure, respectively.

Hydrostaticity	Pressure (GPa)	Mode (cm^−1^)	d*ω*/d*P* (cm^−1^ GPa^−1^)	***γ_i_***
Non-hydrostatic environment	1.2–10.4	T_2g_ (323.5)	4.23	1.07
	10.4–11.9	A_1g_ (175.8)	0.70	0.29
		A_3g_ (215.4)	6.56	2.25
		B_1g_ (248.6)	8.90	2.65
		T_2g_ (371.5)	36.80	7.33
		A_2g_ (371.5)	36.80	7.33
		B_2g_ (410.9)	15.86	2.86
	11.9–20.5	A_1g_ (176.4)	0.66	0.28
		A_3g_ (224.5)	1.15	0.38
		B_1g_ (261.9)	2.55	0.72
		A_2g_ (426.7)	6.62	1.15
		B_2g_ (434.7)	5.02	0.86
Helium	0.5–7.5	T_2g_ (322.5)	7.21	1.83
	7.5–13.9	A_1g_ (173.9)	0.60	0.26
		A_3g_ (226.5)	0.34	0.11
		B_1g_ (249.7)	1.89	0.56
		A_2g_ (370.2)	2.24	0.45
		T_2g_ (370.2)	2.24	0.45
		B_2g_ (405.6)	2.45	0.46
	13.9–20.4	A_1g_ (176.4)	0.51	0.21
		A_3g_ (227.8)	1.05	0.34
		B_1g_ (261.8)	1.77	0.50
		A_2g_ (426.3)	3.06	0.53
		B_2g_ (435.8)	6.64	1.13

Note: The frequencies listed in parentheses correspond to the observed Raman peak positions at the initial pressure of each pressure interval.

**Table 2:** Comparison of the pressure-induced structural phase transition of fluorite under conditions of different pressure-transmitting media and atmospheric temperature.

Methods	Pressure Medium	P_tr_ (GPa)	Refs
Raman spectroscopy	NPT	10.4	This study
	Silicone oil	9.6	
	Mixture of methanol and ethanol (4:1 volume ratio)	8.9	
	Helium	7.5	
Electrical conductivity	NPT	10.5	This study
Raman spectroscopy	Mixture of methanol and ethanol (4:1 volume ratio)	8.5	[10]
	Mixture of methanol and ethanol (4:1 volume ratio)	8.7	[11]
	Argon	9.6	[12]
First-principles calculations	NPT	10.2	[38]

**Table 3:** The fitted parameters of the Arrhenius relation for the electrical conductivity of fluorite within 323–873 K at representative pressures of 7.0, 8.1, 9.3 GPa.

Phase	P (GPa)	T (K)	Log*_σ_*_0_ (S cm^−1^)	Δ*H* (eV)
*α*	7.0	323–573	–1.59 ± 0.11	0.11 ± 0.02
*γ*	7.0	723–873	–0.32 ± 0.09	0.85 ± 0.13
*α*	8.1	323–523	–1.73 ± 0.05	0. 14 ± 0.01
*γ*	8.1	623–873	–0.45 ± 0.11	0. 87 ± 0.04
*α*	9.3	323–473	–0.95 ± 0.14	0.21 ± 0.05
*γ*	9.3	573–873	–0.68 ± 0.12	0.39 ± 0.05

## Data Availability

The data that support the findings of this study are available from the corresponding author upon reasonable request.

## Supplementary Materials

The following supporting information can be downloaded at: https://www.mdpi.com/article/10.3390/molecules31122078/s1. Figure S1: Experimental assemblage for high-temperature and high-pressure electrical conductivity measurements on fluorite; Figure S2: Representative Raman spectral deconvolution results of fluorite at three characteristic pressure points during compression under (a) non-hydrostatic condition and (b) hydrostatic condition using helium as the PTM; Figure S3: Representative Raman spectral deconvolution results of fluorite at three characteristic pressure points during decompression under (a) non-hydrostatic condition and (b) hydrostatic condition using helium as the PTM; Figure S4: High-pressure Raman spectra of fluorite under hydrostatic condition using ME as the pressure-transmitting medium; Figure S5: High-pressure Raman spectra of fluorite under hydrostatic condition using silicone oil as the pressure-transmitting medium; Figure S6: High-pressure Raman spectra of fluorite under hydrostatic condition upon decompression using ME as the pressure-transmitting medium; Figure S7: High-pressure Raman spectra of fluorite under hydrostatic condition upon decompression using silicone oil as the pressure-transmitting medium; Figure S8: Pressure-dependent Raman shifts of fluorite under hydrostatic condition; Figure S9: Representative Nyquist diagram of impedance spectra of fluorite measured at a given pressure of 8.1 GPa over the temperature range of 323–873 K; Figure S10: Representative Nyquist diagram of impedance spectra of fluorite measured at a given pressure of 9.3 GPa over the temperature range of 323–873 K; Table S1: Pressure-dependent Raman shift (d*ω*/d*P*, cm^−1^ GPa^−1^) for fluorite under conditions of silicone oil and ME; Table S2: Theoretical structural parameters of CaF_2_ at selected pressures.

## References

[1] De Leeuw N.H., Cooper T.G. (2003). A computational study of the surface structure and reactivity of calcium fluoride. J. Mater. Chem..

[2] Rubinstein N.A., Zappettini E.O. (2015). Origin and age of rift related fluorite and manganese deposits from the San Rafael Massif, Argentina. Ore Geol. Rev..

[3] Ge X., Guo Q.F., Wang Q.Q., Li T., Liao L.B. (2022). Mineralogical characteristics and luminescent properties of natural fluorite with three different colors. Materials.

[4] Gabitov R.I., Price J.D., Watson E.B. (2005). Solubility of fluorite in haplogranitic melt of variable alkalis and alumina content at 800°–1000 °C and 100 MPa. Geochem. Geophys. Geosyst..

[5] Taguta J., Teme K.C., Ngobeni P. (2020). The role of gangue mineralogy on flowsheet development in fluorite processing. Minerals.

[6] Gao Z.Y., Wang C., Sun W., Gao Y.S., Kowalczuk P.B. (2021). Froth flotation of fluorite: A review. Adv. Colloid Interface Sci..

[7] Mao M., Simandl G.J., Spence J., Marshall D., Simandl G.J., Neetz M. (2015). Fluorite trace-element chemistry and its potential as an indicator mineral: Evaluation of LA-ICP-MS method. Symposium on Strategic and Critical Materials Proceedings.

[8] Huang L.M., Zeng Q., Hu L., Hu Y.H., Zhong H., He Z.G. (2019). The contribution of long-term static interactions between minerals and flotation reagents for the separation of fluorite and calcite. Minerals.

[9] Strzelecki A.C., Migdisov A., Boukhalfa H., Sauer K., McIntosh K.G., Currier R.P., Williams-Jones A.E., Guo X.F. (2022). Fluocerite as a precursor to rare earth element fractionation in ore-forming systems. Nat. Geosci..

[10] Kourouklis G.A., Anastassakis E. (1989). Pressure-induced phase transitions and anharmonicity study of alkaline-earth fluorides. Phys. Status Solidi B.

[11] Speziale S., Duffy T.S. (2002). Single-crystal elastic constants of fluorite (CaF_2_) to 9.3 GPa. Phys. Chem. Miner..

[12] Zhang X.Y., Li L., Yu Y.X., Zhang Q.C., Sun N.Y., Mao Z., Zhang D.Z. (2024). High pressure–temperature study of MgF_2_, CaF_2_, and BaF_2_ by Raman spectroscopy: Phase transitions and vibrational properties of AF_2_ difluorides. ACS Omega.

[13] Hong M.L., Dai L.D., Hu H.Y., Zhang X.Y. (2022). Pressure-induced structural phase transitions in natural kaolinite investigated by Raman spectroscopy and electrical conductivity. Am. Mineral..

[14] Hong M.L., Dai L.D., Hu H.Y., Zhang X.Y., Li C. (2022). High-temperature and high-pressure phase transition of natural barite investigated by Raman spectroscopy and electrical conductivity. Front. Earth Sci..

[15] Zhang X.Y., Dai L.D., Hu H.Y., Hong M.L., Li C. (2025). Constraints on the spin-state transition of siderite from laboratory-based Raman spectroscopy and electrical conductivity under high temperature and high pressure. Geosci. Front..

[16] Klotz S., Chervin J.-C., Munsch P., Marchand G.L. (2009). Hydrostatic limits of 11 pressure transmitting media. J. Phys. D Appl. Phys..

[17] Hu H.Y., Dai L.D., Li H.P., Hui K.S., Sun W.Q. (2017). Influence of dehydration on the electrical conductivity of epidote and implications for high-conductivity anomalies in subduction zones. J. Geophys. Res. Solid Earth.

[18] Yang L.F., Dai L.D., Li H.P., Hu H.Y., Hong M.L., Zhang X.Y., Liu P.F. (2021). High-pressure investigations on the isostructural phase transition and metallization in realgar with diamond anvil cells. Geosci. Front..

[19] Zhang X.Y., Dai L.D., Hu H.Y., Li C. (2023). Pressure-induced reverse structural transition of calcite at temperatures up to 873 K and pressures up to 19.7 GPa. Minerals.

[20] Hu H.Y., Yin C.Y., Dai L.D., Lai J.H., Chen Y.Q., Wang P.F., Zhu J.L., Han S.B. (2024). The role of *α−β* quartz transition in fluid storage in crust from the evidence of electrical conductivity. J. Geophys. Res. Solid Earth.

[21] Liu H.Y., Zhu Q., Yang X.Z. (2019). Electrical conductivity of fluorite and fluorine conduction. Minerals.

[22] Berrada M., McFall A., Chen B. (2025). Raman match: Application for automated identification of minerals from Raman spectroscopy data. Am. Mineral..

[23] Tsuda H., Jongebloed W.L., Stokroos I., Arends J. (1993). Combined Raman and SEM study on CaF_2_ formed on/in enamel by APF treatments. Caries Res..

[24] Liu Y., Guo Q.F., Liu L.Y., Zhang S.X., Li Q.L., Liao L.B. (2023). Comparative study on gemological and mineralogical characteristics and coloration mechanism of four color types of fluorite. Crystals.

[25] Zhang J.C., Luu T.T. (2025). Probing photoinduced structural phase transitions via nonlinear spectroscopy. ACS Photonics.

[26] Koniakhin S.V., Utesov O.I., Yashenkin A.G. (2024). Raman peak shift and broadening in crystalline nanoparticles with lattice impurities. Diam. Relat. Mater..

[27] Vojta T., Hoyos J.A. (2010). Magnetic Grüneisen ratio of the random transverse-field ising chain. Phys. Status Solidi B.

[28] Tripoliti E.K., Thomson A.R., Dobson D.P., Schofield P.F., Wood I.G. (2025). The thermal expansion of the clinopyroxene and garnet polymorphs of Na_2_MgSi_5_O_12_ determined by X-ray powder diffraction. Phys. Chem. Miner..

[29] Musfeldt J.L., Singh S., Smith K.A., Xu X.H., Cheong S.W., Liu Z.X., Vanderbilt D., Rabe K.M. (2025). Pressure-driven polar orthorhombic to tetragonal phase transition in hafnia at room temperature. Chem. Mater..

[30] Dorfman S.M., Jiang F.M., Mao Z., Kubo A., Meng Y., Prakapenka V.B., Duffy T.S. (2010). Phase transitions and equations of state of alkaline earth fluorides CaF_2_, SrF_2_, and BaF_2_ to Mbar pressures. Phys. Rev. B.

[31] Dai L.D., Li H.P., Hu H.Y., Shan S.M. (2008). Experimental study of grain boundary electrical conductivities of dry synthetic peridotite under high-temperature, high-pressure, and different oxygen fugacity conditions. J. Geophys. Res. Solid Earth.

[32] Hu H.Y., Dai L.D., Li H.P., Sun W.Q., Li B.S. (2018). Effect of dehydrogenation on the electrical conductivity of Fe-bearing amphibole: Implications for high conductivity anomalies in subduction zones and continental crust. Earth Planet. Sci. Lett..

[33] Dai L.D., Hu H.Y., Li H.P., Wu L., Hui K.S., Jiang J.J., Sun W.Q. (2016). Influence of temperature, pressure, and oxygen fugacity on the electrical conductivity of dry eclogite, and geophysical implications. Geochem. Geophys. Geosyst..

[34] Dai L.D., Wu L., Li H.P., Hu H.Y., Zhuang Y.K., Liu K.X. (2016). Evidence of the pressure-induced conductivity switching of yttrium-doped SrTiO_3_. J. Phys. Condens. Matter.

[35] Rodewald S., Fleig J., Maier J. (2001). Microcontact impedance spectroscopy at single grain boundaries in Fe-Doped SrTiO_3_ polycrystals. J. Am. Ceram. Soc..

[36] Nagels P., Devreese J., Denayer M. (1964). Electronic conduction in single crystals of uranium dioxide. J. Appl. Phys..

[37] Smith J.S., Serge D., Tse J.S., Sun J., Klug D.D., Ohishi Y. (2009). High-pressure structures and vibrational spectra of barium fluoride: Results obtained under nearly hydrostatic conditions. Phys. Rev. B.

[38] Cazorla C., Errandonea D. (2014). Superionicity and polymorphism in calcium fluoride at high pressure. Phys. Rev. Lett..

[39] Moin P.B. (2012). Thermodynamic potentials and Clausius–Clapeyron equation for strained solids. Phase Transit..

[40] Gerward L., Olsen J.S., Steenstrup S., Malinowski M., Åsbrink S., Waskowska A. (1992). X-ray diffraction investigations of CaF_2_ at high pressure. J. Appl. Crystallogr..

[41] Boulfelfel S.E., Zahn D., Hochrein O., Grin Y., Leoni S. (2006). Sublattice melting by pressure: Superionic conduction in low-dimensional phase interfaces. Z. Anorg. Allg. Chem..

[42] Hu H.Y., Li H.P., Dai L.D., Shan S.M., Zhu C.M. (2011). Electrical conductivity of albite at high temperatures and high pressures. Am. Mineral..

[43] Hu H.Y., Li H.P., Dai L.D., Shan S.M., Zhu C.M. (2013). Electrical conductivity of alkali feldspar solid solutions at high temperatures and high pressures. Phys. Chem. Miner..

[44] Hu H.Y., Dai L.D., Li H.P., Jiang J.J., Hui K.S. (2014). Electrical conductivity of K-feldspar at high temperature and high pressure. Miner. Petrol..

[45] Hu H.Y., Dai L.D., Sun W.Q., Wang M.Q., Jing C.X. (2022). Constraints on fluids in the continental crust from laboratory-based electrical conductivity measurements of plagioclase. Gondwana Res..

[46] Oberschmidt J., Lazarus D. (1980). Ionic conductivity, activation volumes, and frequency-dependent conductivity in crystals with the fluorite structure. Phys. Rev. B.

[47] Soni H.R., Gupta S.K., Talati M., Jha P.K. (2011). Ground state and lattice dynamical study of ionic conductors CaF_2_, SrF_2_ and BaF_2_ using density functional theory. J. Phys. Chem. Solids.

[48] Voronin B.M., Volkov S.V. (2001). Ionic conductivity of fluorite type crystals CaF_2_, SrF_2_, BaF_2_, and SrCl_2_ at high temperatures. J. Phys. Chem. Solids.

[49] Chakraborty S. (1995). Diffusion in silicate melts. Rev. Mineral. Geochem..

[50] Matzke H.J. (1970). Fluorine self-diffusion in CaF_2_ and BaF_2_. J. Mater. Sci..

[51] Wu X., Qin S., Wu Z. (2006). First-principles study of structural stabilities, and electronic and optical properties of CaF_2_ under high pressure. Phys. Rev. B.

[52] Cui S.X., Feng W.X., Hu H.Q., Feng Z.B., Wang Y.X. (2009). Structural stabilities, electronic and optical properties of CaF_2_ under high pressure: A first-principles study. Comput. Mater. Sci..

[53] Khunur M.M., Risdianto A., Mutrofin S., Prananto Y.P. (2012). Synthesis of fluorite (CaF_2_) crystal from gypsum waste of phosphoric acid factory in silica gel. Bull. Chem. React. Eng. Catal..

[54] Suturin S.M., Fedorov V.V., Korovin A.M., Valkovskiy G.A., Tabuchi M., Sokolov N.S. (2021). Controllable CaF_2_ nanosized stripe arrays on Si (001) studied by X-ray and electron diffraction. Surfaces.

[55] Gao R., Li H.P., Zhao J.T. (2015). Dependence of R fluorescence lines of rubies on Cr^3+^ concentration at various temperatures, with implications for pressure calibrations in experimental apparatus. Am. Mineral..

[56] Dai L.D., Pu C., Li H.P., Hu H.Y., Liu K.X., Yang L.F., Hong M.L. (2019). Characterization of metallization and amorphization for GaP under different hydrostatic environments in diamond anvil cell up to 40.0 GPa. Rev. Sci. Instrum..

[57] Perdew J.P., Burke K., Wang Y. (1996). Generalized gradient approximation for the exchange-correlation hole of a many-electron system. Phys. Rev. B.

[58] Peng H., Perdew J.P. (2017). Rehabilitation of the Perdew-Burke-Ernzerhof generalized gradient approximation for layered materials. Phys. Rev. B.

[59] Monkhorst H.J., Pack J.D. (1976). Special points for Brillouin-zone integrations. Phys. Rev. B.

