# Supplementary material for: Hydrostaticity-Sensitive Structural Phase Transition and High-Pressure Phase Diagram in Fluorite: Evidence of Raman Spectroscopy and Electrical Conductivity
Source: Molecules. 2026 Jun 13;31(12):2078. doi: 10.3390/molecules31122078 (PMC13305933; doi:10.3390/molecules31122078)
Supplement: Supplementary file 1 [file molecules-31-02078-s001.zip › molecules-4352874-supplementary.pdf]

Supplemental Material for

Hydrostaticity-sensitive structural phase transition and high-pressure phase diagram in fluorite: Evidence of Raman spectroscopy and electrical conductivity

Mingyu Wu[1,3], Lidong Dai[2*], Haiying Hu[2*], Meiling Hong[1,3] and Chuang Li[1,3]

AFFILIATIONS

[1] Key Laboratory of High-Temperature and High-Pressure Study of the Earth's Interior, Institute of Geochemistry, Chinese Academy of Sciences, Guizhou 550081, China;

[2] School of Physics and Electronic Science, Guizhou Normal University, Guiyang 550025, Guizhou, China

[3] University of Chinese Academy of Sciences, Beijing 100049, China

[*]Authors to whom correspondence should be addressed:

dailidong@gznu.edu.cn and huhaiying@gznu.edu.cn

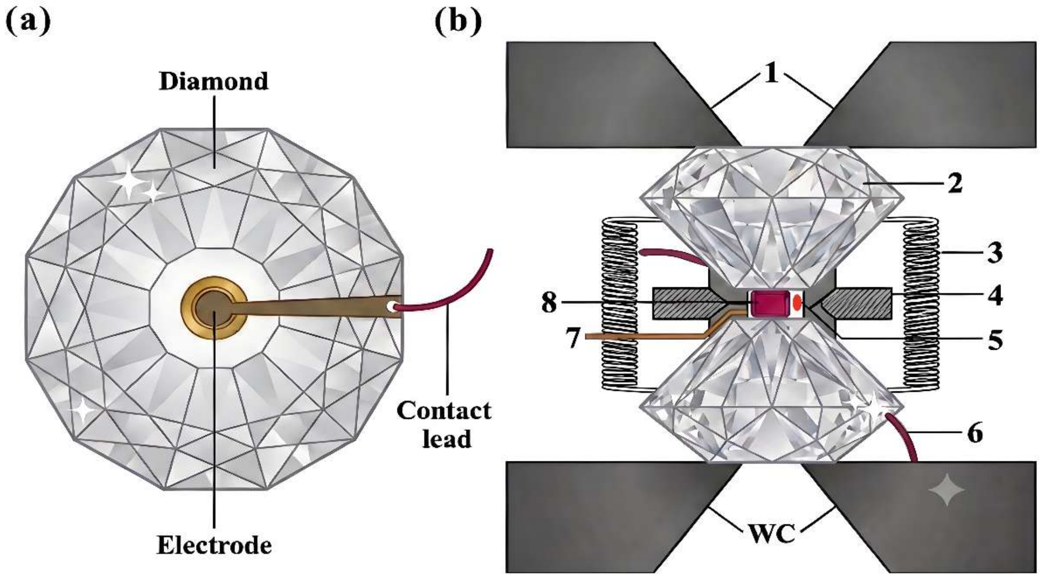

Figure S1. Experimental assemblage for high-temperature and high-pressure electrical conductivity measurements on fluorite. (a) Configuration of plate electrodes installed on the two diamond anvils. (b) Cross-sectional schematic of the externally heated diamond anvil cell (DAC) used for high-temperature and high-pressure electrical conductivity experiments, where (1) tungsten carbide seat; (2) diamond; (3) heating furnace; (4) gasket; (5) ruby; (6) electrode; (7) thermocouple; (8) sample.

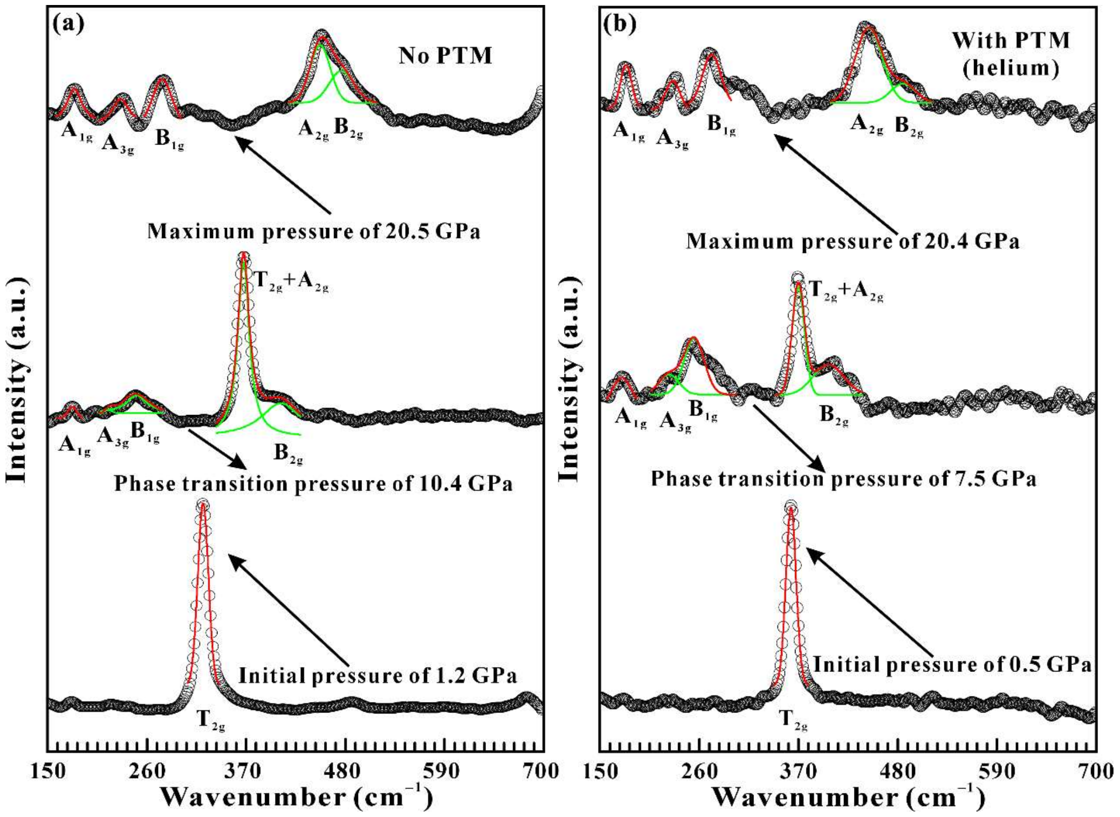

Figure S2. Representative Raman spectral deconvolution results of fluorite at three characteristic pressure points during compression under (a) non-hydrostatic condition and (b) hydrostatic condition using helium as the PTM. The three pressure points correspond to the initial pressure, the phase transition pressure, and the maximum pressure, respectively. Open circles represent the experimental data, red curves denote the fitted envelope, and green curves represent the individual Gaussian peak components. The vibrational modes are labeled accordingly.

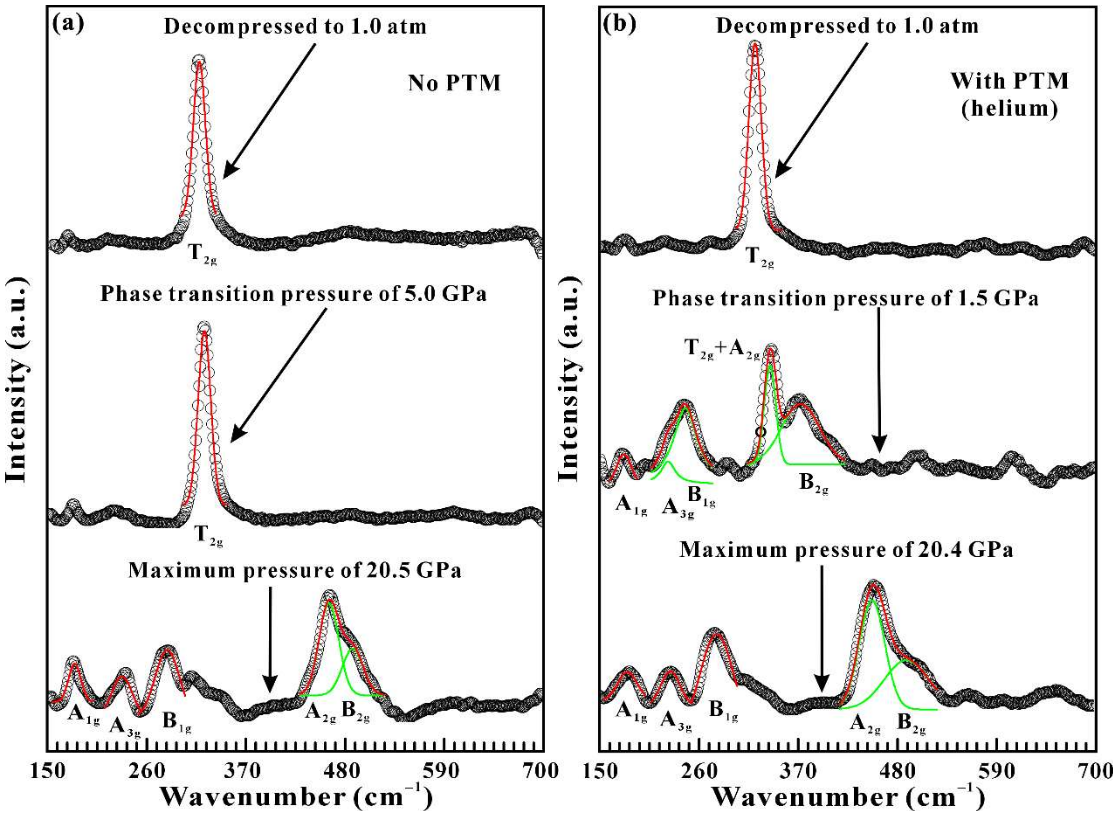

Figure S3. Representative Raman spectral deconvolution results of fluorite at three characteristic pressure points during decompression under (a) non-hydrostatic condition and (b) hydrostatic condition using helium as the PTM. The three pressure points correspond to the maximum pressure, the phase transition pressure, and ambient pressure, respectively. Open circles represent the experimental data, red curves denote the fitted envelope, and green curves represent the individual Gaussian peak components. The vibrational modes are labeled accordingly.

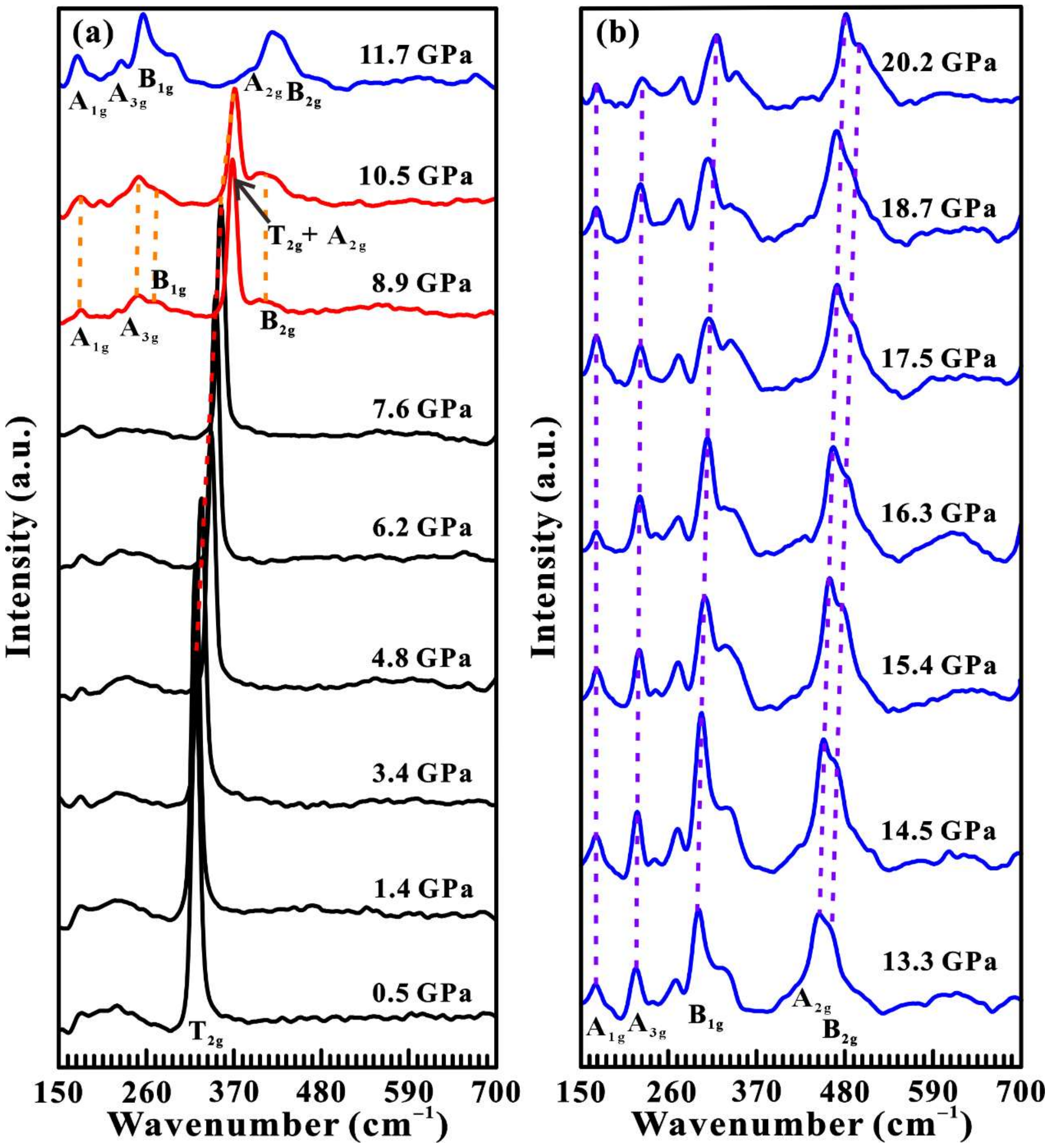

Figure S4. High-pressure Raman spectra of fluorite under hydrostatic condition using ME as the pressure-transmitting medium. (a) Spectra from 0.5 to 11.7 GPa and (b) from 13.3 to 20.2 GPa.

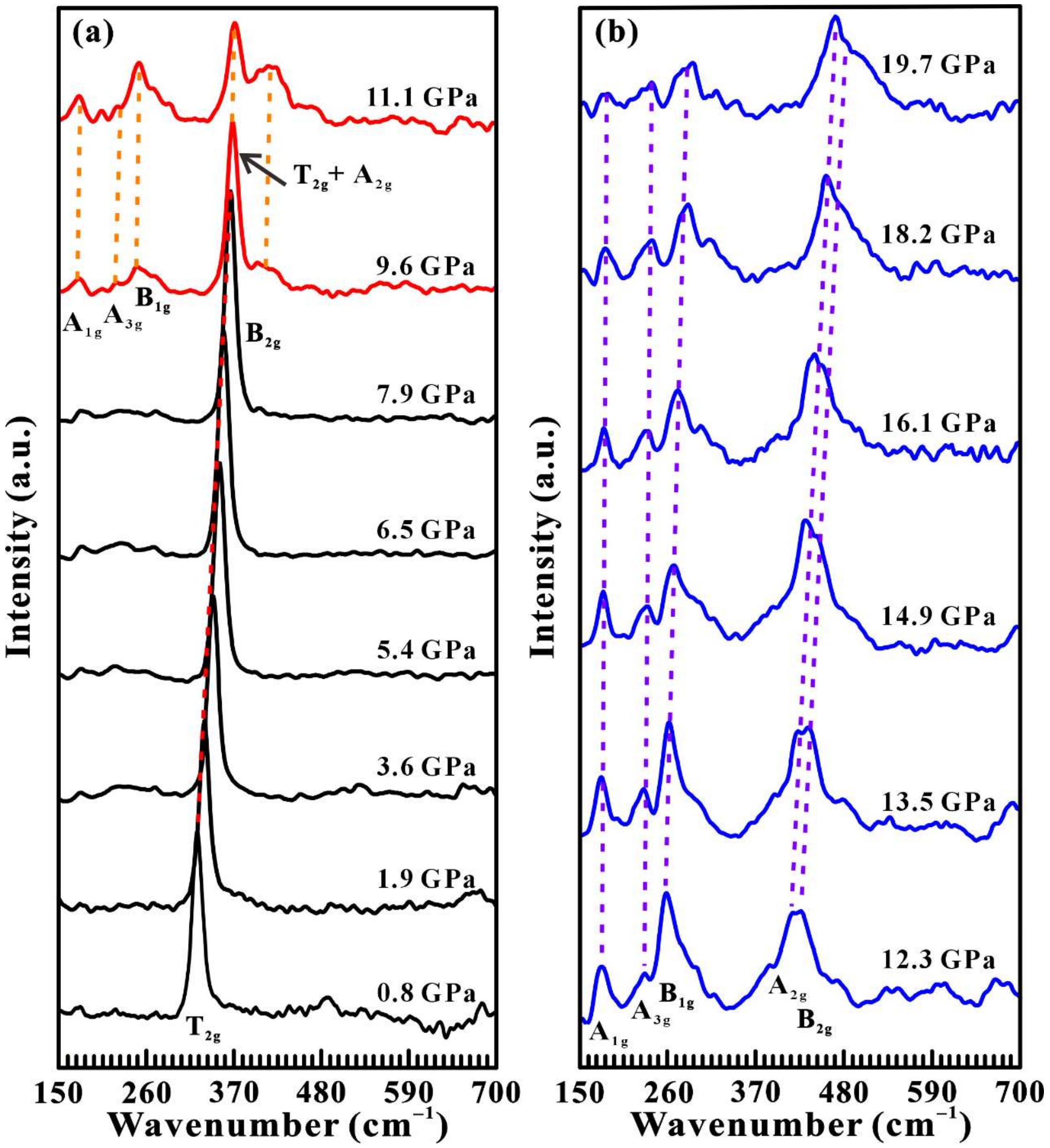

Figure S5. High-pressure Raman spectra of fluorite under hydrostatic condition using silicone oil as the pressure-transmitting medium. (a) Spectra from 0.8 to 11.1 GPa and (b) from 12.3 to 19.7 GPa.

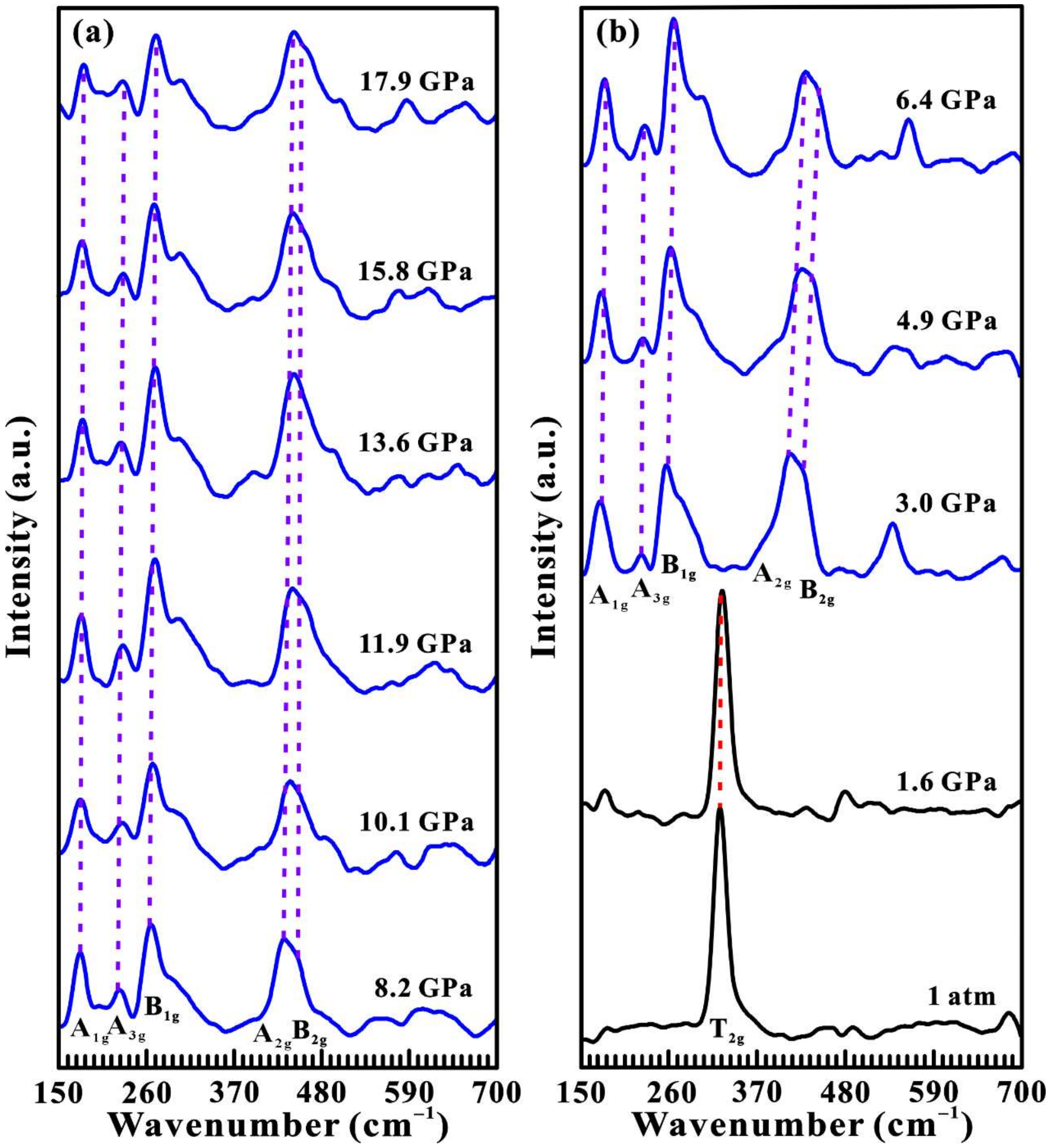

Figure S6. High-pressure Raman spectra of fluorite under hydrostatic condition upon decompression using ME as the pressure-transmitting medium. (a) Spectra from 17.9 to 8.2 GPa and (b) from 6.4 GPa to 1 atm.

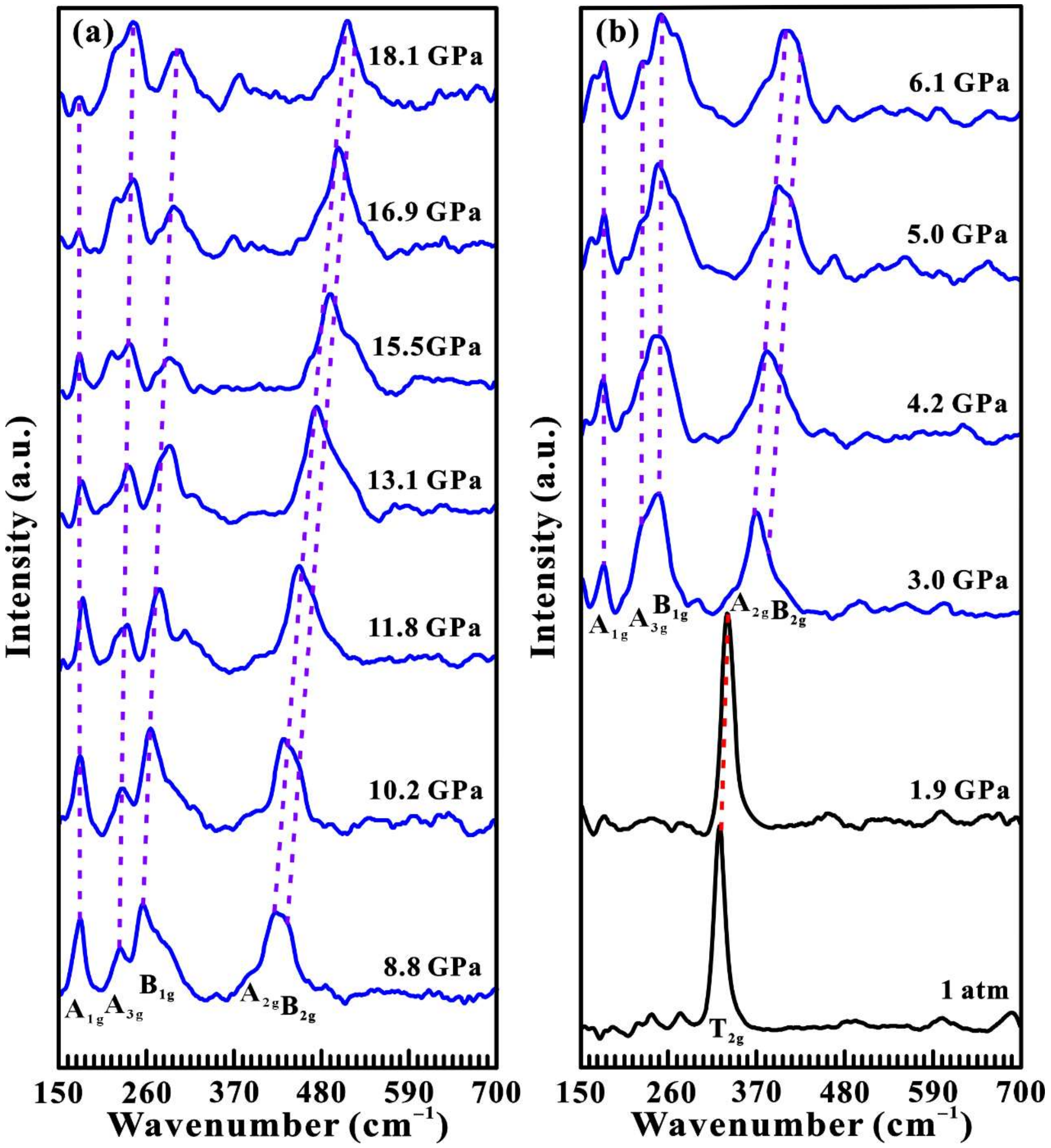

Figure S7. High-pressure Raman spectra of fluorite under hydrostatic condition upon decompression using silicone oil as the pressure-transmitting medium. (a) Spectra from 18.1 to 8.8 GPa and (b) from 6.1 GPa to 1 atm.

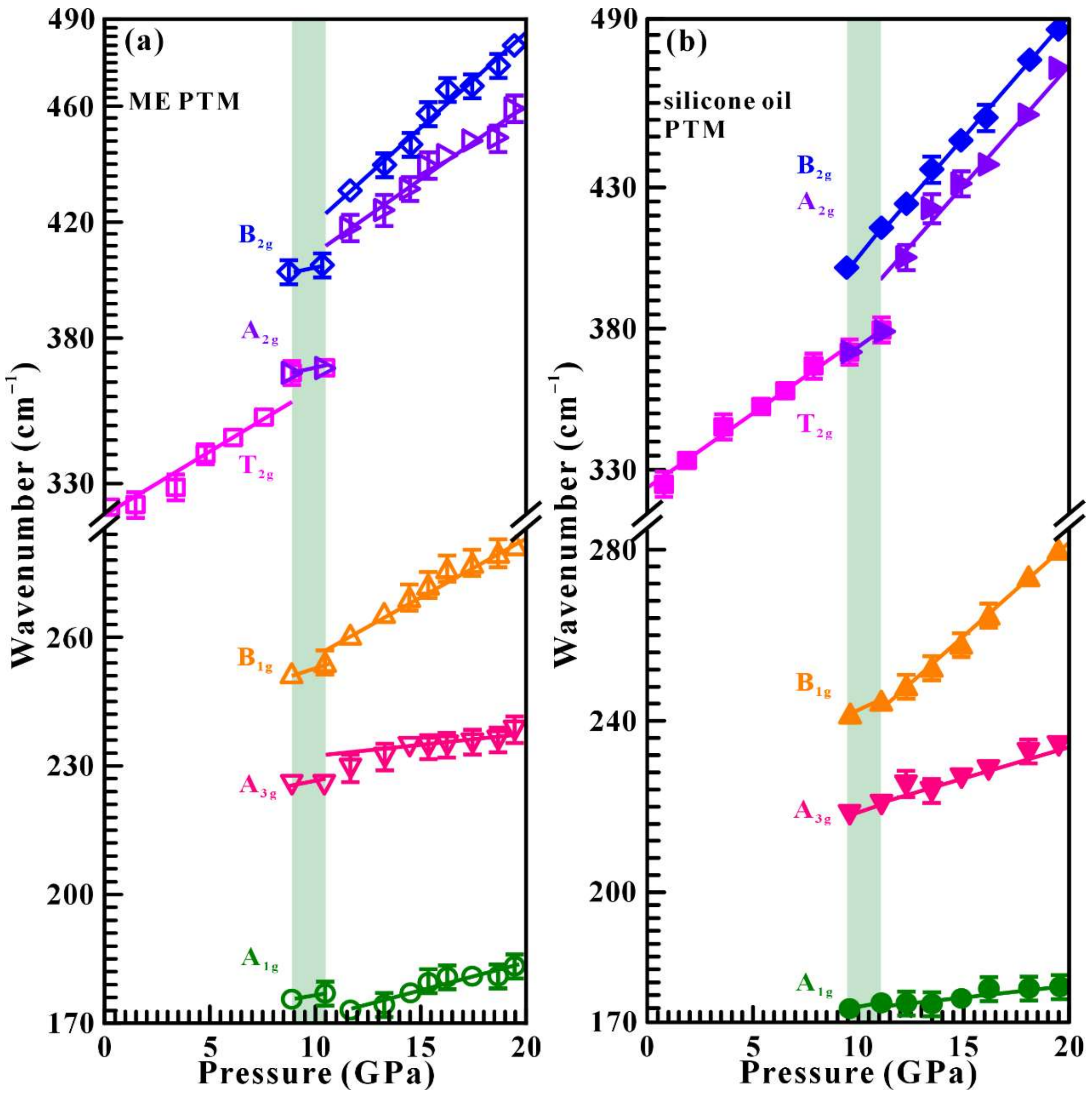

Figure S8. Pressure-dependent Raman shifts of fluorite under hydrostatic condition: (a) hydrostatic condition using ME as the pressure-transmitting medium and (b) hydrostatic condition using silicone oil as the pressure-transmitting medium, respectively.

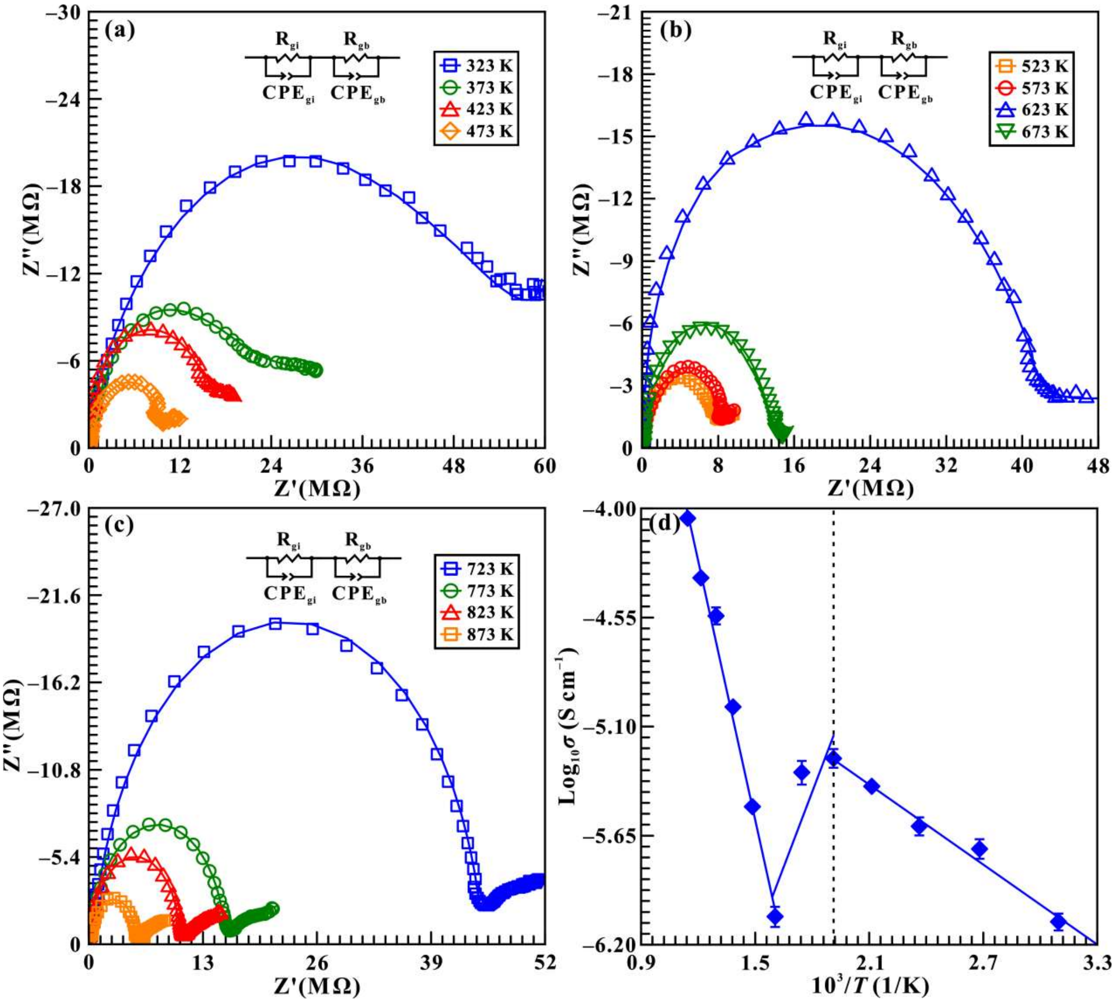

Figure S9. Representative Nyquist diagram of impedance spectra of fluorite measured at a given pressure of 8.1 GPa over the temperature range of 323–873 K. (a) 323–473 K; (b) 523–673 K; (c) 723–873 K; (d) Logarithmic plot of electrical conductivity versus temperature for fluorite. Solid and dashed lines are provided as visual guides.

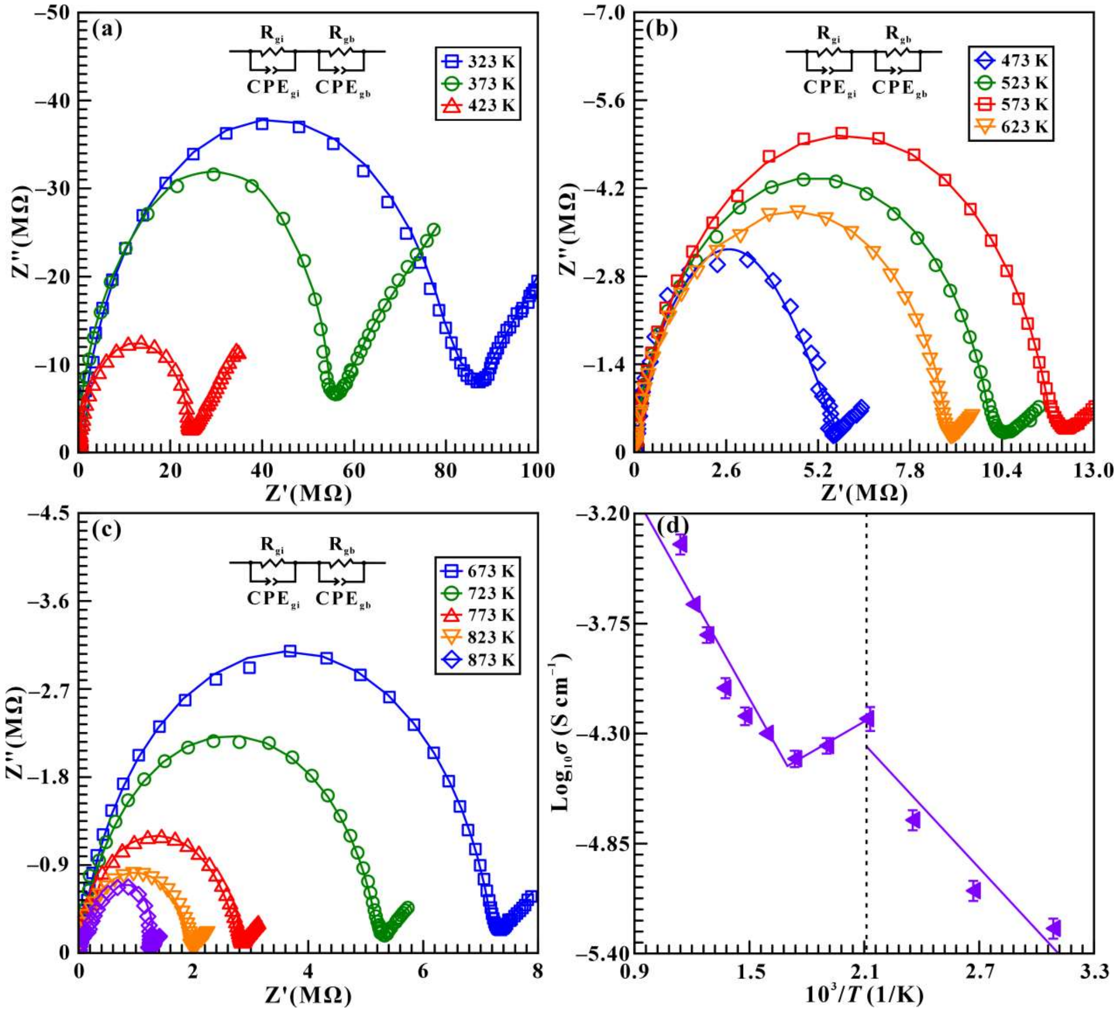

Figure S10. Representative Nyquist diagram of impedance spectra of fluorite measured at a given pressure of 9.3 GPa over the temperature range of 323–873 K. (a) 323–423 K; (b) 473–623 K; (c) 673–873 K; (d) Logarithmic plot of electrical conductivity versus temperature for fluorite. Solid and dashed lines are provided as visual guides.

Table S1. Pressure-dependent Raman shift (dω/dP, cm^{-1} GPa^{-1}) for fluorite under conditions of silicone oil and ME. Here, ω (cm^{-1}) and P (GPa) represent the Raman wavenumber and pressure, respectively.

Hydrostaticity	Pressure (GPa)	Mode (cm^{-1})	dω/dP (cm^{-1} GPa^{-1})	γ_i
Silicone oil	0.8–9.6 GPa	T_{2g} (324.6)	5.34	1.35
	9.6–11.1 GPa	A_{1g} (174.0)	0.97	0.41
		A_{3g} (222.9)	1.63	0.54
		B_{1g} (248.5)	2.43	0.72
		T_{2g} (371.5)	5.37	1.07
		A_{2g} (371.5)	5.37	1.07
		B_{2g} (401.6)	9.36	1.72
	12.3–19.7 GPa	A_{1g} (175.3)	0.60	0.38
		A_{3g} (230.2)	1.53	0.39
		B_{1g} (256.0)	4.73	0.72
		A_{2g} (405.2)	8.41	1.14
		B_{2g} (423.9)	8.69	0.86
ME	0.5–8.9 GPa	T_{2g} (322.5)	4.43	1.13
	8.9–10.5 GPa	A_{1g} (173.1)	0.76	0.33
		A_{3g} (223.2)	0.07	0.03
		B_{1g} (247.5)	1.56	0.47
		A_{2g} (368.2)	1.50	0.30
		T_{2g} (368.2)	1.50	0.30
		B_{2g} (402.8)	1.51	0.28
	11.7–20.2 GPa	A_{1g} (175.5)	1.23	0.52
		A_{3g} (226.9)	0.92	0.30
		B_{1g} (256.1)	3.09	0.89
		A_{2g} (418.3)	5.05	0.89
		B_{2g} (431.4)	6.56	1.13

Note: The frequencies listed in parentheses correspond to the observed Raman peak positions at the initial pressure of each pressure interval.

Table S2. Theoretical structural parameters of CaF_2 at selected pressures.

Pressure (GPa)	Phase	a (Å)	b (Å)	c (Å)
0 GPa	$Fm\bar{3}m$	5.521	–	–
10 GPa	*Pnma*	3.512	5.851	6.900
20 GPa	*Pnma*	3.338	5.678	6.776
